# Heavy episodic drinking in adolescence and alcohol-related problems in adulthood: A developmental approach to alcohol use across the life course

**DOI:** 10.1017/S0954579422001249

**Published:** 2022-12-12

**Authors:** Gemma T. Wallace, Corey Whichard, Megan Augustyn, Kimberly L. Henry

**Affiliations:** 1 Department of Psychology, Colorado State University, Fort Collins, CO, USA,; 2 School of Criminal Justice, University at Albany, State University of New York, Albany, NY, USA; 3 Department of Criminology & Criminal Justice, The University of Texas at San Antonio, San Antonio, TX, USA

**Keywords:** adolescent alcohol use, binge drinking, equifinality, heavy episodic drinking, multifinality

## Abstract

Heavy episodic drinking (HED) is a major public health concern, and youth who engage in HED are at increased risk for alcohol-related problems that continue into adulthood. Importantly, there is heterogeneity in the onset and course of adolescent HED, as youth exhibit different trajectories of initiation and progression into heavy drinking. Much of what is known about the etiology of adolescent HED and alcohol-related problems that persist into adulthood comes from studies of predominantly White, middle-class youth. Because alcohol use and related problems vary by race/ethnicity and socioeconomic status, it is unclear whether previous findings are relevant for understanding developmental antecedents and distal consequences of adolescent HED for minoritized individuals. In the current study, we utilize a developmental psychopathology perspective to fill this gap in the literature. Using a racially and economically diverse cohort followed from adolescence well into adulthood, we apply group-based trajectory modeling (GBTM) to identify patterns of involvement in HED from age 14 to 17 years. We then investigate developmental antecedents of GBTM class membership, and alcohol-related distal outcomes in adulthood (~ age 31 years) associated with GBTM class membership. Results highlight the importance of adolescent alcohol use in predicting future alcohol use in adulthood.

## Introduction

Heavy episodic drinking (HED), commonly defined as consuming five or more alcoholic drinks for males or four or more drinks for females in one sitting, is a prevalent concern among adolescents, with 4.1% of individuals aged 12–17 in the US reporting past-month HED in 2020 (Substance Abuse and Mental Health Services Administration [SAMHSA], 2021). HED has been associated with numerous immediate and long-term problems, including alcohol-related accidents and acute toxicity (Chung et al., 2018), alcohol dependence (Kuntsche et al., 2017), mental and physical health concerns (Fortier et al., 2021), interpersonal relationship difficulties (Fairbairn et al., 2018), and increased likelihood of legal difficulties (Walters, 2014), among others. Compared to other age groups, adolescents have heightened risk for HED and associated concerns; rates of HED tend to increase across adolescence before peaking in young adulthood (Chung et al., 2018; National Institute on Drug Abuse, 2022). Importantly, and consistent with the expectations of developmental psychopathology (Cicchetti & Rogosch, 2002), there is heterogeneity in adolescent HED. Only a portion of adolescents engage in HED, and some do so more frequently and consistently than others (Donovan & Molina, 2013). Further, younger age at onset for HED has been associated with poorer outcomes, likely due to impacts of substance use during a critical period of brain development (Mewton et al., 2020; Squeglia & Gray, 2016). Individual differences in HED patterns and age at onset can have implications for alcohol use across adolescence that can extend into adulthood (Chung & Jackson, 2019).

Assessment of the origins and course of HED during adolescence, as well as the outcomes of observed behavioral patterns extending into adulthood, is prerequisite to the development of effective intervention and prevention initiatives. Drawing from the developmental psychopathology perspective, equifinality and multifinality are key tenets of the development and course of harmful substance use (Cicchetti & Rogosch, 1996). Equifinality occurs when different antecedents predict the same behavioral outcome, while multifinality occurs when the same antecedents confer vulnerability to multiple different outcomes (Cicchetti & Rogosch, 1996; Mayes & Suchman, 2006). Equifinality and multifinality posit that a harmful developmental trajectory (e.g., HED during adolescence) can be caused via many different paths, and that same harmful trajectory can lead to different outcomes (Cicchetti & Rogosch, 1996). Informed by this framework, intervention programs may be more effective if they are designed to account for similarities and differences in the developmental circumstances that lead to adolescent HED. Likewise, assessment of the prevention and intervention of alcohol use disorders in adulthood may be more effective if the course of alcohol use during adolescence is considered. Through a comprehensive and thorough assessment of the etiological factors leading up to HED, the course of HED, and the ultimate outcomes of HED – we may increase the likelihood of identifying mechanisms for preventing or intervening in adverse developmental trajectories of alcohol use.

In this study, we aim to make a significant contribution to the literature by applying a developmental psychopathology framework to HED during adolescence (Cicchetti, 1999). Using a rich data source comprised of 939 adolescents interviewed fourteen times over eighteen years, we model the onset and escalation of HED over the course of adolescence (using eight equally spaced measures from age 14 to 17). We then examine key antecedents of discerned HED trajectories extending from birth to early adolescence and across a variety of domains of a child’s life (e.g., family, school, peer). In addition, we assess the long-term consequences of patterns of adolescent HED on adult alcohol use and related problems. Our ability to investigate this longitudinal course using a single cohort is relatively unique and marks a notable contribution to the existing literature. Further, the cohort study used in this examination is unique in its own right – some 85% of the sample is non-White (68% African American, 17% Hispanic, and 15% White), and more than half of the families were considered low socioeconomic status at the start of the study.

### Investigation of alcohol trajectories during adolescence

Since the 1990s, numerous studies have examined heterogeneity in adolescent alcohol use using group-based trajectory modeling (GBTM) (see reviews by Chung et al., 2018; Chung & Jackson, 2019). GBTM is a longitudinal and person-centered statistical method, and in the current application, GBTM is capable of discerning probabilistic subgroups of individuals based on their reported HED patterns over time (Nagin & Odgers, 2010a). Most GBTM studies on adolescent HED have identified three to five trajectories, including abstainers and/or infrequent engagers in HED, as well as subgroups of individuals who engage in HED that are distinguished by HED age at onset and frequency (Brunborg et al., 2018; Chassin et al., 2002; Hill et al., 2000; Oesterle et al., 2004; Tucker et al., 2003). Notably, most GBTM studies on adolescent HED have used samples that are primarily composed of White middle-class participants. Fewer studies have examined alcohol use trajectories in samples of adolescents with significant representation of Black youth or adolescents from socioeconomically disadvantaged backgrounds, and these studies have generally used alcohol use measures other than HED to discern subgroups (e.g., D’Amico et al., 2016; Finlay et al., 2012; Flory et al., 2006; Hill et al., 2000). Assessment of HED trajectories in racially minoritized groups is needed, as much of our current knowledge of these issues is garnered from predominantly White samples, and it is inappropriate to assume that underlying mechanisms and patterns function the same way across all groups (Chung & Jackson, 2019; Gatzke-Kopp, 2016).

The limited focus on longitudinal patterns of HED among Black and Hispanic populations is particularly salient given that racially minoritized individuals experience greater ill effects of substance use. Although rates of alcohol use tend to be higher among White adolescents compared to Black and Hispanic youth (Miech et al., 2020; Pamplin et al., 2020), racially minoritized individuals have greater risk of HED in adulthood (Pamplin et al., 2020) and of experiencing negative consequences if they drink (Witbrodt et al., 2014; Zapolski et al., 2014). Black and Hispanic adults experience disproportionately high rates of alcohol-related problems in adulthood in the United States compared to White adults, including more alcohol-related physical health conditions and less access to and utilization of treatment for alcohol use disorders (e.g., Chartier et al., 2014; Hadland & Baer, 2014; Mulia et al., 2017; Zapolski et al., 2014; Zemore et al., 2018). Taken together, there is a critical need to examine the etiology, course, and consequences of HED among representative samples of adolescents, including racially and economically minoritized individuals (Zemore et al., 2018).

### Causes and consequences of heavy episodic drinking trajectories during adolescence

Identifying HED trajectories can provide information about temporal relations between antecedents to HED involvement and outcomes in adulthood (e.g., Brunborg et al., 2018; Chassin et al., 2002). To date, numerous antecedents have been implicated in adolescent HED (see reviews by Chung et al., 2018; Chung & Jackson, 2019). In alignment with the social-ecological framework, factors influencing alcohol use include individual-level factors nested within home, school and work, and community environment factors (Sudhinaraset et al., 2016). Thus, it is advantageous to consider factors from each of these salient life domains of adolescents’ lives to elucidate risk for HED. Moreover, the timing of antecedents across development is important (Chung & Jackson, 2019). Alcohol use patterns can have roots in both proximate antecedents, such as current socio-environmental factors and beliefs and attitudes (Kuntsche et al., 2017), as well as in distal biopsychosocial antecedents from earlier stages of development, beginning as early as *in utero* (e.g., Goldschmidt et al., 2019). The life span developmental perspective posits that alcohol use patterns, risk factors for use, and outcomes from use vary across stages of development, and distal factors can sometimes influence proximate factors over time (Schulenberg & Maggs, 2008). It is therefore beneficial to consider experiences from across development when predicting adolescent HED trajectories. However, most studies examining antecedents to HED trajectories examined risk/protective factors from relatively narrow windows of time.

In the present study we use both the social-ecological and lifespan developmental frameworks to select and evaluate potential antecedents to adolescent HED trajectories, comprising factors that have previously been associated with adolescent alcohol use and bridge multiple life domains and stages of development. We examine demographic characteristics of adolescents and their parents/caregivers, distal antecedents (early life adversity, mental health, and behavioral problems), and proximate antecedents from multiple life domains, including environmental opportunities to access alcohol, social and family environment factors (peer and parental alcohol use), and beliefs (religiosity, school attachment, and beliefs about underage drinking) (Brown et al., 2001; Chung & Jackson, 2019; Henry & Slater, 2007; Rossow et al., 2016; Yuen et al., 2020). To our knowledge, these factors have not been simultaneously examined as antecedents to adolescent HED trajectories. Since these antecedents encompass multiple domains of risk and span the developmental period from *in utero* to adolescence, consideration of this salient collection of antecedents may shed important light on the etiology of HED trajectories.

Adolescent HED has also been associated with increased risk for alcohol-related problems in adulthood (Chung et al., 2018). HED trajectories that are characterized by younger age at onset (Tucker et al., 2005) and more frequent and/or heavier HED (Olsson et al., 2016) have predicted more severe alcohol use and related issues in adulthood. For example, HED trajectories that involved more frequent adolescent HED predicted higher rates of alcohol use disorder when participants were in their early to mid-20s (Brunborg et al., 2018; Chassin et al., 2002). Alcohol-related problems in adulthood have also been associated with alcohol use age at onset and frequency during adolescence, even if severe alcohol use did not occur in adolescence (Chung & Jackson, 2019). To date, most longitudinal studies of HED that employ GBTM have relied on designs that followed participants into their early to mid-20s. Limited, if any, research has examined relations between HED trajectories in adolescence and alcohol-related problems that extend beyond emerging adulthood (ages 18–25 years) and into early adulthood (ages 26–39 years), especially among racially diverse samples of youth (Santrock, 2009). While alcohol-related problems typically develop in adolescence and emerging adulthood, alcohol use disorders have the highest prevalence in individuals aged 18–29 years, and 90% of individuals who develop alcohol use disorders do so by their late 30s (American Psychiatric Association, 2013). Extending studies of outcomes beyond emerging adulthood and into participants’ 30s may increase understanding of nuanced relations between HED in adolescence and subsequent alcohol use across a wider developmental period with elevated risk for alcohol-related problems.

### A developmental psychopathology perspective to HED trajectories

A developmental GBTM approach can be used to evaluate equifinality and multifinality of HED trajectories during adolescence, as well as their impacts on alcohol-related problems in adulthood. The principle of equifinality is used in this context to examine the developmental pathways that can lead to adolescent HED, while the principle of multifinality is used to examine the extent to which HED during adolescence can lead to different styles of alcohol consumption in adulthood. Assessment of our research questions through the lens of equifinality and multifinality can offer important insights and shed new light on poorly understood phenomena. For example, it is unclear if the same combinations of risk and protective factors across development predict equal likelihood of one HED trajectory over another, and if different trajectories are uniquely associated with alcohol use tendencies in adulthood. This latter issue is particularly salient to a developmental psychopathology perspective, as understanding the prevalence and circumstances of continuity and discontinuity of behavior is critical (Schulenberg et al., 2014). If distinct or shared etiologies are identified for different HED trajectories and subsequent alcohol-related problems in adulthood, this could inform tailored prevention and intervention approaches to reduce the burden of alcohol-related problems in specific populations (Chung & Jackson, 2019).

GBTM and other latent trajectory methodologies have been used to generate a great deal of knowledge about the course of HED and other forms of alcohol use, as well as key antecedents and consequences of these behavioral patterns. In the present study, we build on this extensive research base to address three overarching research aims. First, we employ GBTM to identify distinct trajectories of HED from age 14 through age 17 using a racially/ethnically and socioeconomically diverse cohort of adolescents (Research Aim 1). Second, we augment the GBTM solution identified in Aim 1 to consider antecedents of trajectory membership, focusing on a comprehensive array of antecedents spanning from birth to early adolescence (Research Aim 2). Third, we determine if GBTM class membership is predictive of alcohol-related distal outcomes in adulthood (Research Aim 3). Thus, our study synthesizes existing work to develop a comprehensive, person-centered model of HED, and HED’s antecedents and consequences, in an ethnically/racially and socioeconomically diverse cohort of adolescents followed longitudinally for a period of 17 years.

## Methods

### Data

#### Rochester Youth and Development Study

The Rochester Youth Development Study (RYDS) is a prospective cohort study of 1,000 youth who were enrolled in either the seventh or eighth grade in the Rochester, New York public school system in 1988. We provide a brief overview of the study here (see Thornberry et al. [2001] for more details). The primary aim of RYDS was to study the development of adolescent antisocial behaviors; thus, the study oversampled males and recruited adolescents with a probability proportional to the arrest rate within their census tracts in 1986. The average age at enrollment was 13.6 years and 73% of the adolescents were male. Sixty-eight percent were African American, 17% Hispanic, and 15% were White. Adolescents (G2s) and their primary caregivers (G1s) were interviewed semiannually from 1988 to 1992 (phase 1; waves 1–9) and annually from 1994 to 1996 (phase 2; waves 10–12). G2 participants were additionally interviewed biannually from 2003 to 2006 (phase 3; waves 13–14). Official data were collected from schools, the police, and social services through Phase 3. All data collection procedures were approved by the University at Albany Institutional Review Board.

### Measures

Given the measurement space of RYDS’ interviews and relatively large sample of youth, our analyses included a wide selection of measures. In the text below we limit our focus to measures included in the final models. We provide more details on select variables and describe other measures considered for analyses in the Appendix.

#### G2 heavy episodic drinking during adolescence

To measure G2 involvement in HED, we constructed binary indicators from seven waves of RYDS interview data (wave 2 to wave 8) collected at approximate 6-month intervals, which encompasses ages 14 through 17 for the average respondent. Beginning in wave 2, G2 respondents were asked: “Since we interviewed you last time, did you drink beer, wine, or hard liquor without your parents’ permission?” If the respondent answered “yes,” they were asked: “Since the last interview, did you drink beer, wine, or liquor at least once a month?” If the respondent answered “yes,” they were then asked to provide details on the amount of beer, wine, and hard liquor that they *usually* consumed, with separate questions for each type of alcohol (e.g., “When you drink beer, how much do you usually drink?”). Following the guidelines established by the National Institute on Alcohol Abuse and Alcoholism (2021), we treated a “single drink” as 12 ounces of beer, 5 ounces of wine, or 1.5 ounces of hard liquor. We defined “heavy episodic drinking” as consuming at least 4–5 drinks in one sitting.^1^

*Heavy episodic drinking* was a binary variable coded 1 if the G2 respondent drank alcohol at least once a month since the last interview and usually consumed at least 4–5 drinks per sitting, and 0 otherwise (the item was set to missing if the respondent did not complete the alcohol consumptions items). We applied this coding scheme to each of the seven utilized waves of RYDS data (i.e., seven binary indicators of HED for each respondent across waves 2–8).

#### G2 demographics

We constructed measures of basic demographic characteristics for G2 (adolescent) respondents. *G2 Female* is a binary indicator coded 1 for females and 0 for males. We created two binary indicators (*G2 White vs. G2 Black*, *G2 Hispanic* vs. *G2 Black*) based on G2’s self-reported race/ethnicity. We treated Black G2 respondents as the reference category. *G2 Age at baseline* is a continuous measure of how old the respondent was in years when they enrolled in RYDS. *G2 Grade at baseline* is a binary indicator of grade at the start of RYDS, coded 1 for seventh grade and 0 for eighth grade. *G2 Community arrest rate*, a stratifying variable for sample composition, is a continuous measure representing the residential arrest rate for the home address of each G2 at the start of RYDS based on Rochester police record data for 1986.

#### Distal antecedents

##### Early life adversity.

We included measures of whether *G2*’*s biological mother drank alcohol while pregnant with G2* (1 = drank while pregnant, 0 = did not drink while pregnant) and the *age of G2*’*s biological mother when G2 was born* (in years). These variables were constructed from G1 interviews at wave 12, which were administered to G1 about 9 years after the first RYDS interview. We also include a retrospective measure of *G2 experienced neglect* <*age 12* (1 = experienced neglect, 0 = did not experience neglect), which was drawn from G2’s wave 12 interview when the average G2 respondent was around 23 years old. See Appendix for more details.

##### Early problems.

During the wave 1 interview, when G2 respondents were about age 13.5, G2 respondents were asked whether they had ever drunk beer, wine, or hard liquor without a parent’s permission, and “How old were you the first time you did this?” *G2 drank alcohol* <*age 12* is a binary indicator of whether G2 reported they drank alcohol without their parents’ permission for the first time before age 12 (1 = drank prior to age 12, 0 = did not drink prior to age 12). In the wave 12 interview, G1 was asked series of binary questions related to G2’s mental health. G1 was asked whether G2 ever displayed “anxiety attacks or nervousness,” “depression,” “schizophrenia or other mental illnesses,” or “other behavioral or emotional problems.” If G1 responded “yes” to any item, G1 was then asked how old was G2 when this was first noticed. If G1 reported that any of these four items were first observed in G2 before age 12, then *G2 mental health symptoms* <*age 12* was coded as 1. Otherwise, if G1 reported that G2 never experienced any of these four items or first displayed these symptoms after age 12, this variable was coded as 0 (the item was set to missing if this scale was not completed).

#### Proximate antecedents

##### Beliefs.

All measures of beliefs come from the G2 wave 1 interview. G2 respondents provided their attitudes toward alcohol use in response to the following question: “How wrong do you think it is for kids like you to drink alcohol?” *G2 agrees that drinking alcohol is wrong* ranged from 1 (“Not wrong at all”) to 4 (“Very wrong”). G2 respondents self-reported their level of religiosity with “How much do you think of yourself as a religious person?”. *G2 religiosity* ranged from 1 (“Not at all”) to 5 (“Very much”). Finally, G2 respondents were asked a variety of questions regarding their attitudes and experiences related to school. We used these data to create a measure G2’s overall *school attachment*, with higher scores reflecting greater levels of attachment. See Appendix for more details.

##### Opportunity factors.

Analyses also included measures that approximated G2’s ability to obtain and consume alcohol. All opportunity measures were generated from the G2 wave 2 interview. Given the relationship between unstructured socializing with peers and alcohol use (Osgood et al., 1996), we included a measure of *G2 time with peers without adult supervision*. This ordinal measure was based on G2’s response to: “Think of a usual week during the school year. How often do you and your friend(s) get together where no adults are present?” Responses ranged from 1 (“Never”) to 5 (“Every day”). *G1 supervision of G2* is based on two items asking G2 “How often does G1 [primary caregiver] know where you are?” and “How often would G1 [primary caregiver] know who you are with when you are away from home?”. Responses ranged from 1 (“Never”) to 4 (“Often”). G2 supervision is the mean of these two items. *G2 perceived access to alcohol* is based on their response to the following question: “How easy would it be for you to buy alcohol or to have someone else buy it for you?” Values ranged from 1 (“Very difficult”) to 4 (“Very easy”).

##### Social context.

Analyses also include measures of G2’s social context that likely promote alcohol use. Measures were taken from the wave 2 interviews. *G1 frequency of heavy drinking* is an ordinal variable based on G1’s response to: “When drinking, how often do you have as many as 3 or 4 drinks?” Values ranged from 0 (“Never”) to 3 (“Nearly every time”). If G1 was a nondrinker, we coded them 0. G2 reported their perception of their friends’ alcohol use in response to: “How many of your friends drank alcohol?” Response options ranged from 1 (“None of them”) to 4 (“Most of them”), and *G2 friend alcohol use* is an ordinal measure based on the responses to this question.

#### G2 alcohol use in adulthood

We operationalized G2 alcohol use in adulthood via six variables based on questions from the wave 14 interview, when respondents were approximately 31 years old. *Drank alcohol at least once per month* is a binary indicator based on G2’s response to: “During the past year, did you drink beer, wine, wine coolers, or liquor at least once a month?” *Binge drinking* is operationalized as consuming 5 or more drinks in one sitting (CDC, 2022). It is based on the yes/no response to: “During the past year, have you had five or more drinks at one sitting?” *Frequency of binge drinking* is a count variable that represents how G2 answered the follow-up question: “About how many times have you done this during the past year?” *Drunkenness* is a binary indicator based on G2’s response to: “During the past year, have you gotten drunk?” *Frequency of drunkenness* is a count variable that reflects G2’s estimate of how often this happened during the past year. *Alcohol-related problems* is a binary indicator based on whether G2 answered “yes” to at least one of a series of 10 statements about whether they experienced various life problems in the past year as a direct result of drinking alcohol. See Appendix for more details.

### Missing data

Tables 4 and 6 report the level of missingness for each covariate included in our analyses. Rather than drop participants with missing values on our antecedents and adult measures of alcohol use, we used multiple imputation to retain these observations (Little & Rubin, 2019). Specifically, we used multiple imputation by chained equations (MICE) in Stata 17 (StataCorp, 2021) to appropriately account for the level of measurement for each antecedent and outcome and produce 20 imputed datasets. Where noted, models were fit across the 20 imputed datasets, and the results were combined to produce a final set of estimates using the procedures outlined by Rubin (1987).

### Analytic strategy

#### Research aim #1: identifying latent drinking trajectories

Our first analysis stage aimed to discern heterogeneity in HED during adolescence (spanning age 14 to age 17) using group-based trajectory modeling (GBTM; Nagin, 2005). GBTM uses finite mixture models to identify probabilistic clusters of individuals who display similar individual-level trajectories or patterns of HED over “time.” Specifically, GBTM identifies unobserved latent trajectories of HED, with each trajectory representing the average HED trend for individuals with highest probability of belonging to that group (Brame et al., 2001; for a comprehensive review, see Nagin & Odgers, 2010b). GBTM analyses were completed in Stata using the Traj procedure.

Recall that RYDS originally recruited G2 respondents who were in the seventh grade or eighth grade and ranged in age from 12 to 15 years of age; therefore, we estimated the latent trajectories using values of G2 age at each wave as the time measure.^2^ GBTM uses all available data to estimate latent trajectories. Estimated patterns of HED between ages 14 and 17 were observed for 939 G2s who participated in at least three waves of data collection during adolescence and responded to questions about their drinking behavior. We limited the sample to G2s who had at least three time points in order to maximize the amount of relevant data used for the identification of the trajectories and to improve model accuracy.

GBTM estimation begins with setting a specified number of groups and then re-estimating the model until the higher-order terms for each group are significant (Nagin, 2005). Final model selection includes the optimization of the Bayesian Information Criterion (BIC) as well as additional parameters suggested by Nagin (2005): (1) the mixture probabilities are reasonably close to the percentage of the sample assigned (through hard classification) to each group; (2) the 95% confidence intervals for the mixture probabilities are reasonably narrow; (3) the mean posterior probability of classification for each group, which indicates the likelihood that each individual belongs to the assigned group, exceeds .7; (4) the odds of correct classification is close to or exceeds 5. Since our outcome variable was a binary measure of HED, our GBTM used the logit model specification.

#### Research aim #2: identifying antecedents of trajectory group membership

The second stage of our analysis focused on identifying antecedents of trajectory membership. We were interested in how early life circumstances and experiences during childhood/early adolescence may increase or decrease the probability that a G2 respondent would follow a particular HED trajectory. Due to the amount of missing data on our antecedent variables after listwise deletion,^3^ we opted to investigate the correlates of our GBTM results through a “classify-analyze” approach (Jennings, 2015) that would allow for the use of multiply imputed data sets to account for missing data.^4^

Within a latent class framework, Roeder et al. (1999) argued that when the mean posterior probabilities of group membership exceed .7, individuals can be ‘hard-classified’ into the group with the highest posterior probability for the purposes of post-estimation analyses because classification uncertainty is minimal. Because the average posterior probability of each trajectory group in our models was .89 or higher, indicating high classification accuracy, we hard-classified RYDS participants into patterns of HED based on the highest posterior probability of group membership. We then treated membership in the HED trajectories as the dependent variable for this analysis; we assigned respondents to groups based on the results of the GBTM, and then included this observed binary variable as the dependent variable in a logistic regression.

We used the social-ecological and life span developmental approaches to inform our modeling strategy (Windle, 2010). Factors that influence adolescent alcohol use emerge in a developmental sequence and operate across multiple life domains, including individual, family, peer, school, and neighborhood (Sudhinaraset et al., 2016; Wilcox, 2003). Moreover, both distal and proximate antecedents from the various life domains can influence alcohol use across the life course (Chung & Jackson, 2019). In order for variable effects to be observed without adjusting for other antecedents that would be considered causally subsequent, we structured our second stage analysis as a series of hierarchical models. We began with an initial model containing only demographic predictors of HED, then added distal antecedents, and finally added proximate antecedents. Each model was fit across the 20 imputed datasets and the results were combined to produce a final set of point estimates and appropriately adjusted standard errors.

#### Research aim #3: predicting alcohol use in early adulthood

The third and final stage of our analysis examined the extent to which the trajectories of HED during adolescence predicted G2’s alcohol use in adulthood. In other words, we investigated whether HED trajectories from age 14 through age 17 predicted alcohol use assessed at age 31. First, we used the Stata Traj procedure to estimate the point estimate and 95% CI for each early adult alcohol outcome among each trajectory class obtained in the GBTM (Research Aim #1). As the Stata Traj procedure does not allow multiply imputed datasets to be used, we followed up this analysis by again using a classify-analyze approach in which participants were assigned to a trajectory based on their probability of belonging to the latent group in the original GBTM. HED trajectory membership was examined as a predictor of wave 14 alcohol use while controlling for demographics, distal, and proximate antecedents. We used logistic regressions for the categorical outcomes: monthly alcohol use, binge drinking, drunkenness, and problems associated with alcohol use. Negative binomial regressions were used for the count outcomes: frequency of binge drinking and frequency of drunkenness (which displayed evidence of overdispersion). All models were fit across the 20 multiply imputed datasets and the results were combined to produce a final set of point estimates and adjusted standard errors.

## Results

### Group-based trajectory models

We applied GBTM to identify latent trajectories of G2 HED using seven waves of data collected biannually, ranging from when the average G2 respondent was 14 through 17 years old. Table 1 displays the prevalence of HED across each half-year age. Here, we translated the wave-specific measures to age-specific measures. Note that because of age-heterogeneity in when adolescents began the study, as well as the precise timing of interviews, there is more missing data at any given half year than there was in any particular wave (e.g., across all waves the maximum percentage of responses missing was 15%). Table 2 reports the goodness-of-fit statistics for the best-fitting models representing two and three unique groups. Though we estimated models with four or more groups, the results fit the data poorly and generated very small groups (i.e., containing <5% of respondents) and often failed to converge. Based on the selection criteria recommended by Nagin (2005), we selected a two-group model. The two-group model optimized the BIC and AIC scores generated from the Traj program in Stata and achieved the minimum average posterior probability of group membership threshold of .7 for each group. Full diagnostics of the two-group model (Nagin, 2005) are available in Appendix Table A. Table 3 presents the logit coefficients and the estimated and assigned membership proportions for our final two-group model. Since the estimated group membership proportion approximates the assigned group membership proportions, this provides further evidence of adequate model fit (Nagin, 2005, p. 89).^5^

**Figure 1.**
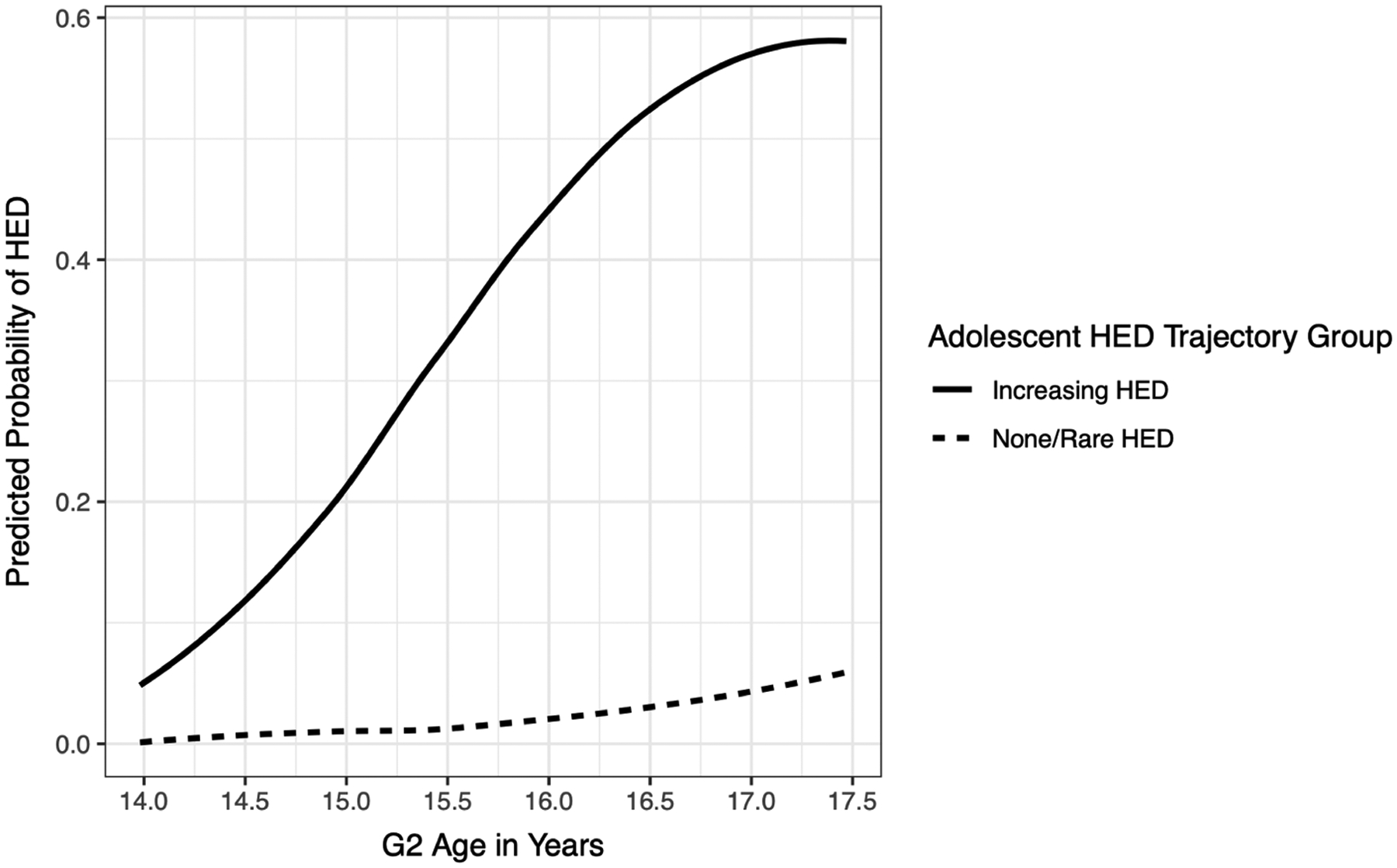
G2 Group trajectories of heavy episodic drinking (HED) during adolescence. The *y*-axis represents the predicted probability that a G2 respondent engaged in HED during the last six months. The *x*-axis depicts G2 age at each time interval. The trend lines represent the observed group means for the estimated trajectories over time.

Figure 1 visualizes the latent classes of HED generated through GBTM. The GBTM discerned two latent groups with distinct patterns of involvement in HED: None or Rare HED and Increasing HED. The None/Rare HED group was estimated to include about 82.5% of the G2 sample. Notably, this group did not completely abstain from HED during adolescence. Rather the probability of HED at any age was near-zero, although this probability began to increase (slightly) after age 16, but still remained below .1. The “Increasing HED” group comprised about 17% of the G2 sample. Among this group, the probability of HED at age 14 years was low, around 10%, and it steadily increased through age 17 years. This group is noteworthy because it exhibited a higher risk of HED involvement at every observed age in adolescence.

### Identifying antecedents of trajectory membership

Given the measurement space of RYDS, there were a large number of theoretically relevant risk and protective factors (i.e., antecedents) available for inclusion in analysis, but model overfitting was a concern (Stolzfus, 2011). Therefore, to identify the most important antecedents to include in our final models, we took the following steps: (1) we limited antecedents to only those that demonstrated a significant bivariate correlation with adolescent HED trajectory group membership; (2) we investigated bivariate correlations between antecedents and, to prevent potential issues of multicollinearity, limited antecedents to only those that did not have a bivariate correlation with another antecedent of ≥.5. Antecedents not included in the final analyses as a result of these steps include multiple demographic variables (G1 employment, G1 marital status, and G1 receipt of welfare benefits); several distal antecedents in the form of early adversity (G2’s mother smoked cigarettes while pregnant, G2 low birth weight) and G2 early problems (G2 cognitive symptoms <age 12, G2 engaged in shoplifting <age 12, G2 engaged in assault <age 12); and multiple proximate antecedents in the form of beliefs (G2 positive relationships with teachers) and social context (number of kids in G2’s neighborhood who drank alcohol, G2 perception of inconsistent punishment). Table 4 presents descriptive statistics for all variables utilized in our second stage of analysis, including retained antecedents as well as adolescent HED trajectory membership. Table 4 provides the descriptive statistics for the full sample, as well as by trajectory class membership. Because of our variable selection strategy, all antecedents (demographics, distal factors, and proximate factors) listed in Table 4 were significantly associated with trajectory class membership, except for community arrest rate. We elected to retain community arrest rate despite its nonsignificant association with trajectory class membership because it was used as a sampling parameter in RYDS.

**Figure 2.**
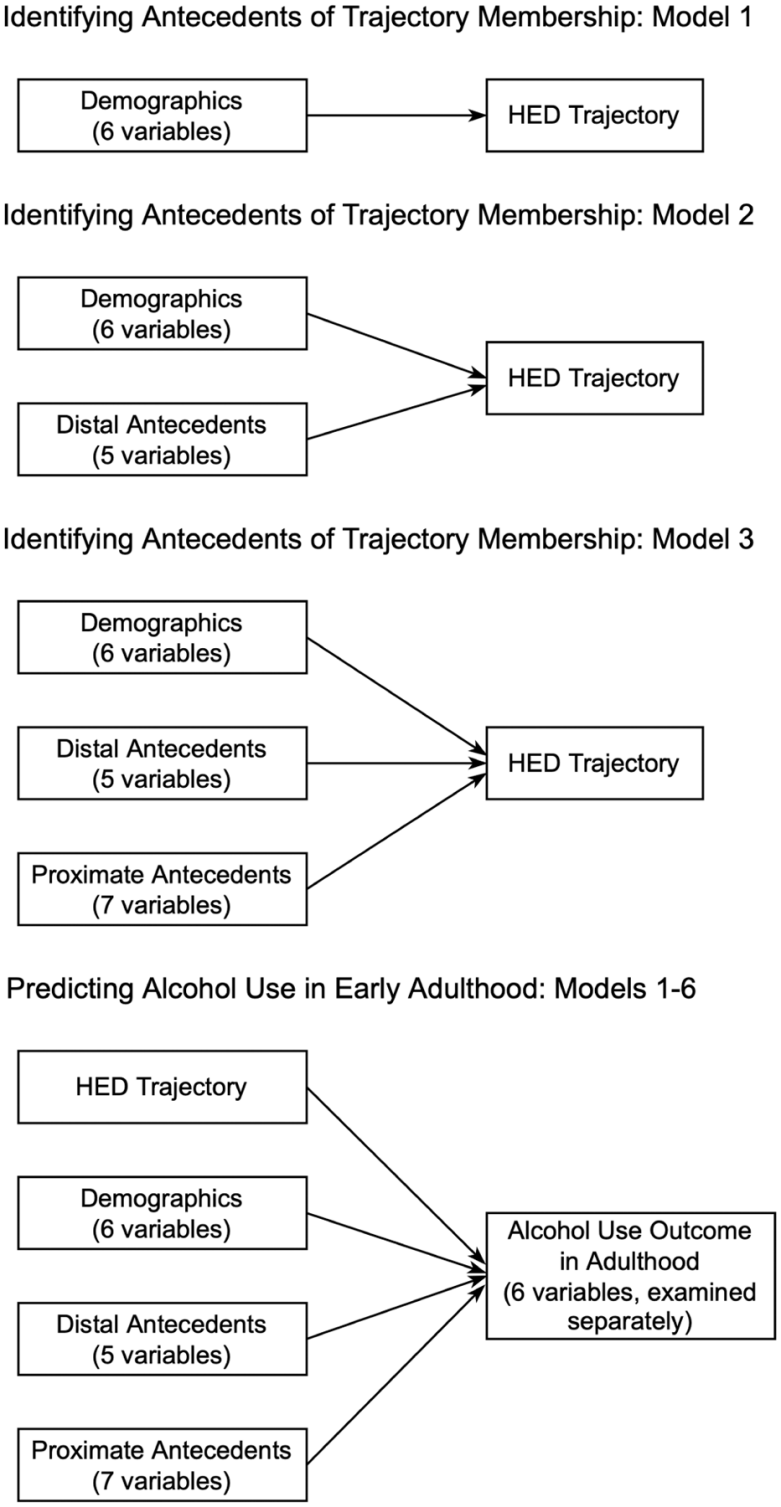
Conceptual models for examining antecedents and distal alcohol use outcomes of HED trajectory membership using a classify-analyze approach. Accompanying model results are presented in Tables 5 and 7.

Table 5 presents the results of the logistic regression models predicting G2 membership in the Increasing HED group (relative to the None/Rare HED trajectory group) generated from 20 multiply imputed data sets. Figure 2 presents the conceptual models for these analyses. We started with a baseline model containing demographics and then expanded the analysis to include distal antecedents of adolescent HED and then proximate antecedents of adolescent HED. In the demographic model (Model 1 of Table 5), sex was the only significant predictor of HED trajectory membership. Females were less likely to be classified as “Increasing HED”. When distal antecedents were added as predictors (Model 2 of Table 5), all three early life adversity factors (i.e., G1 drinking alcohol while pregnant with G2, G2 experiencing neglect prior to the age of 12, and G1 younger age at G2 birth) were associated with an increased likelihood that G2 was classified in the Increasing HED group. In addition, one early problem behavior, G2 alcohol use prior to age 12, was associated with an increased likelihood of classification in the Increasing HED group (adjusting for all other variables in the model). Finally, Model 3 of Table 5 added the proximate antecedents. Several variables added additional predictive power to the model. Lower school attachment, weaker beliefs in the wrongfulness of alcohol use, greater access to alcohol, more frequent G1 engagement in heavy drinking, and perceptions that more friends of G2 used alcohol were all associated with an increased likelihood that G2 would be classified in the Increasing HED trajectory class.

### Predicting alcohol use in early adulthood

**Figure 3.**
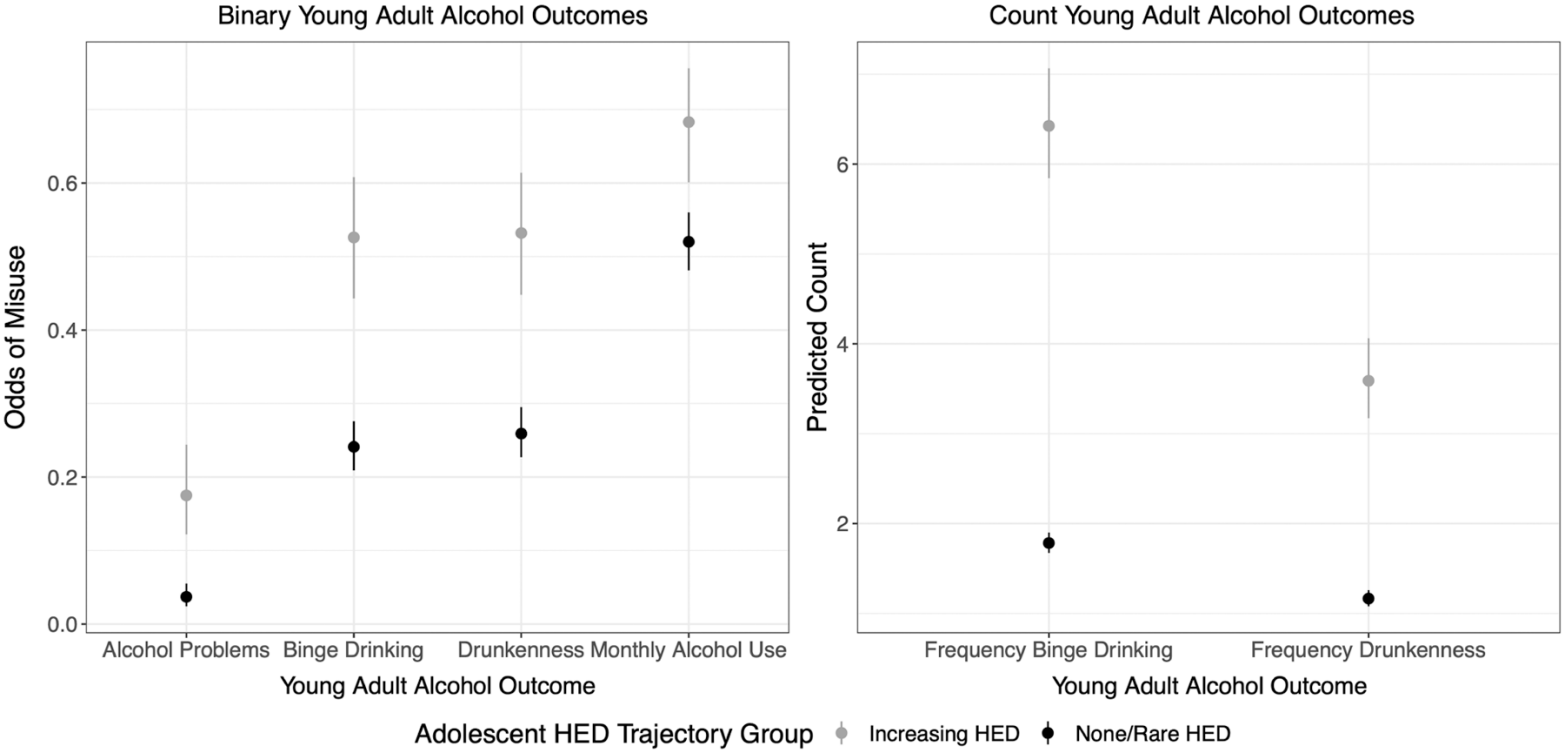
Fitted estimates of the young adult alcohol outcomes (and their 95% CI) across latent trajectory classes within the GBTM.

Table 6 presents descriptive statistics for the adult alcohol outcome variables in the full sample and by the two HED trajectories based on most likely class membership. Figure 3 presents the fitted estimates of the young adult alcohol outcomes (and their 95% CI) as a function of trajectory class membership. These estimates take into account uncertainty in trajectory class membership, but employ list-wise deletion for missing data on the young adult alcohol outcomes (*N* = 758). The figure demonstrates that for all alcohol variables considered, members of the Increasing HED group had a substantially higher average score (odds for binary variables and counts for count variables) as compared to members of the None/Rare HED group. In fact, none of the 95% CI’s overlap when comparing the two trajectory groups.

To determine the extent to which trajectory class membership predicted the young adult outcomes above and beyond the antecedents (i.e., demographics and both the distal and proximate antecedents) considered in Research Aim 2, each of the G2 alcohol use measures collected at wave 14 were regressed on probabilistic HED trajectory group membership and all antecedents. These results utilized the multiply imputed data and are presented in Table 7. Figure 2 visualizes the conceptual models for this analysis step. Holding constant the antecedents, individuals who were classified in the Increasing HED group were more likely to have engaged in binge drinking, been drunk, and experienced problems associated with alcohol use within the past year. More specifically, the odds of binge drinking and drunkenness were each about two times higher, while the odds of experiencing alcohol-related problems were about 2.8 times higher for those classified in the Increasing HED group relative to individuals who were characterized by none or very rare HED during adolescence, adjusting for antecedents. Classification in the Increasing HED group was also positively related to monthly alcohol use, the frequency of binge drinking, and the frequency of drunkenness, although the 95% confidence intervals surrounding the point estimates included 1 (suggesting a nonsignificant effect once the antecedents were controlled).

## Discussion

The current study used interview data from the RYDS to investigate the developmental origins and patterns of HED during adolescence and its connection to problematic drinking in adulthood. We pursued three research aims. First, we applied group-based trajectory modeling (GBTM) to identify distinct patterns in the onset and developmental progression of HED during adolescence. Second, we examined developmental antecedents of HED by regressing probabilistic trajectory membership across a host of reported antecedents from earlier stages of the life course, ranging from when the respondent was *in utero* (retrospectively reported when respondents were young adults) through early adolescence (contemporaneously reported when respondents were early adolescents). Finally, we investigated the extent to which HED trajectories during adolescence predicted problematic alcohol use 17 years later, when the average respondent was 31 years old. We discuss the key findings from each of these research aims in turn.

### Latent trajectories of adolescent HED

Our trajectory analysis discerned two latent groups of HED during adolescence: (1) a very large group comprised of adolescents who did not, or rarely, engaged in HED (i.e., the None/Rare HED group); (2) a smaller group comprised of adolescents whose likelihood of engagement in HED increased substantially over the course of adolescence (i.e., the Increasing HED group). These results are somewhat consistent with research on adolescent alcohol use generally, as well as GBTM studies of adolescent HED in particular. Since the mid-1980s, representative studies of alcohol use have consistently found that the majority of US adolescents do not engage in HED (Chung et al., 2018). Moreover, studies using GBTM to analyze the onset and progression of adolescent HED commonly find that the largest latent group consists of youth who either abstain from or exhibit minimal involvement in HED through age 18 (e.g., Chassin et al., 2002; Hill et al., 2000; Tucker et al., 2003). In the current study, we identified a large latent group (i.e., the None/Rare HED group) that parallels these findings. Around age 14 and the likely transition to high school, alcohol use accelerates and the prevalence of high-risk drinking behavior steadily increases for a substantial minority of youth (Keyes et al., 2012). Similarly, most GBTM studies of adolescent HED have identified a trajectory group where adolescents exhibit little involvement in HED in early adolescence, but become increasingly likely to engage in HED across adolescence (e.g., Chassin et al., 2002). This a group for whom prevention and intervention programming for substance amisuse target their efforts.

It is important to note that in other (predominantly White) samples, additional latent groups that further refine the timing of onset and pattern of HED have been identified (Oesterle et al., 2004; Park et al., 2018). For example, previous studies have identified a third latent HED trajectory characterized by stable, moderate risk for HED across adolescence, as opposed to consistently low or increasing HED over time (see review by Chung et al., 2018). In all likelihood, characteristics of the RYDS sample may have led to this difference and the identification of only two groups. For instance, this sample consists of predominantly racial/ethnic and socioeconomic minorities. It also represents youth originating from one geographic location in the United States with its own context of availability of alcohol and norms for alcohol use. As such, local context may play a significant role in patterns of use. Furthermore, these data were collected from 2006 forward; thus, studies identifying patterns of HED prior to this date may be tapping into differential cohort effects. Although it may seem that two latent groups lack much of the previously observed nuance in patterns of HED, the two-class pattern has important implications for prevention in populations like the RYDS sample. While HED patterns are likely more dimensional than binary for many individuals, in this sample, engagement in any HED conferred risk for subsequent increases in HED. Unlike studies utilizing samples with overrepresentation of more privileged social identities, we did not identify a subgroup who could engage in HED infrequently without the risk of developing more severe HED tendencies. This is particularly informative for programming that targets minoritized social identities, as well as work that seeks to understand the consequences of HED across subgroups of adolescents.

### Developmental antecedents of HED trajectories

Our second stage analysis investigated the precursors to probabilistic HED trajectory membership. We aimed to establish a profile of risk/protective factors that could elucidate the developmental processes that underlie heterogeneity in the onset and course of adolescent HED. We drew on social ecological models of adolescent alcohol use (Wilcox, 2003) by incorporating potential antecedents from multiple social contexts (e.g., peer groups, family, neighborhoods, school). In addition, we drew on the social-ecological and life span developmental perspectives to assess a wide-ranging collection of antecedents spanning from childhood to early adolescence. Importantly, we structured our analyses to evaluate whether distal antecedents (e.g., fetal alcohol exposure and early alcohol use) contributed to adolescent alcohol use after accounting for more proximate antecedents (e.g., association with alcohol-using friends).

Providing support for equifinality, antecedents from a variety of domains and childhood/adolescent stages were identified. When considered in hierarchical models, we found sex (i.e., male) was a key demographic risk factor for membership in the Increasing HED group. Adding distal antecedents to the model identified additional important risk factors for membership in the Increasing HED group. These included risk factors spanning from prenatal experiences (younger age of motherhood and mothers’ alcohol use while pregnant) to late childhood (experiencing neglect and initiating alcohol use prior to age 12). Furthermore, we identified several proximate antecedents to adolescent HED. Higher levels of school attachment, beliefs that alcohol use is wrong during early adolescence, and perceptions of lower ease of accessing alcohol were protective factors against classification in the Increasing HED group. Social context also added predictive power, with parental frequency of heavy drinking and friend alcohol use in early adolescence increasing the likelihood of classification in the Increasing HED group. Many of these risk factors in the RYDS sample have also been identified in studies of adolescent alcohol use utilizing both similarly diverse (e.g., Finlay et al., 2012; Hill et al., 2000) and predominantly White, middle-class samples of adolescents (see reviews by Chung et al., 2018; Chung & Jackson, 2019). Thus, these risk factors appear to be robust across populations of adolescents.

Taken together, results are in line with the lifespan developmental framework positing that distal and proximate antecedents to HED may relate to each other over time. Early adversity can influence development in such a way that subsequent problems across successive stages of the life course become increasingly likely (Moffitt, 1993). Although our sample size precluded evaluating indirect effects between the antecedent variables, antecedents from different stages of development could possibly influence each other, whereby early-life adversity contributes to the development of problems in childhood that – in turn – increase the likelihood of problems in adolescence (e.g., Masten et al., 2005). For example, in utero alcohol exposure has been linked to difficulties in cognitive functioning and difficult temperament (e.g., Spadoni et al., 2007), which are risk factors for childhood emotional and behavioral problems (e.g., Staff et al., 2015). Youth who exhibit early emotional and behavioral problems are at greater risk of using alcohol during adolescence (e.g., Hicks et al., 2010). Moreover, adults who struggle with alcohol use may exhibit lower-quality parenting skills and be more likely to rear their children in adverse household and neighborhood contexts where alcohol is more widely accessible (Donovan, 2019). Compounding things further, children of adults with alcohol-related problems appear more likely to later develop friendships with peers who use alcohol (e.g., Blanton et al., 1997). These processes may be interrelated, resulting in “clusters” of risk factors that jointly contribute to the early initiation and escalation of alcohol use in adolescence (Chung & Jackson, 2019).

Models importantly identified several antecedents that can be malleable to prevention and/or intervention initiatives. For example, respondents classified in the Increasing HED group were more likely to initiate alcohol use in childhood (prior to age 12) and engage in HED at a younger age than those classified in the None/Rare trajectory. Earlier age at onset for alcohol use increases the probability that an adolescent will develop an alcohol use disorder (Hingson et al., 2006) and tends to co-occur with a more rapid escalation of alcohol use (Donovan & Molina, 2013). Thus, programs aimed at delaying the age at onset for alcohol use may protect against adolescent HED (Moure-Rodríguez & Caamano-Isorna, 2020). As another example, results also corroborate a considerable body of evidence that adolescents who engage in HED tend to associate with friends who also engage in HED (e.g., Steinberg, 2008). We measured friend alcohol use based on each respondent’s perception of their friends’ behavior (i.e., descriptive norms). Descriptive norms exhibit a powerful, positive association with adolescents’ own self-reported alcohol use (e.g., Capaldi et al., 2009), likely due to both peer influence and homophily (i.e., the tendency for youth who use alcohol to befriend other youth who also use alcohol) (e.g., Patrick et al., 2014). Promoting pro-social friend selection and altering descriptive norms may therefore reduce risk of adolescent HED (Brooks-Russell et al., 2014; Hoeben et al., 2021; Patrick et al., 2014). Existing evidence-based interventions have been successful at delaying alcohol use age at onset (Hawkins et al., 1992), correcting overestimates of normative perceptions about alcohol use (Faggiano et al., 2010; Lewis & Neighbors, 2006), and promoting pro-social friend selection and interpersonal skills for addressing peer influences on alcohol use (Faggiano et al., 2010; Hawkins et al., 1992). Results support the implementation, or continued implementation, of such interventions in schools and communities similar to those sampled in the RYDS study.

### Alcohol use in adulthood

Our third stage analysis examined how HED trajectories during adolescence predicted alcohol use some 17 years later, when the average RYDS respondent was 31 years old. Consistent with expectations, respondents with probabilistic membership in the None/Rare HED group during adolescence reported substantially lower involvement in alcohol use and related problems in adulthood than respondents with probabilistic membership in the Increasing HED group. It is important to note, however, that many respondents in the None/Rare HED group displayed harmful patterns of drinking in adulthood – for example, about 25% engaged in binge drinking at least once in the past year and about 4% experienced one or more alcohol-related problems. Likewise, most respondents in the Increasing HED group did not display harmful patterns of drinking in adulthood – for example, 51% did not engage in binge drinking and about 84% did not experience alcohol-related problems. This demonstrates that while adolescent patterns of drinking are indeed predictive of drinking styles in adulthood – HED in adolescence in no way dictates that the individual will go on to engage in harmful alcohol use in adulthood. This notion is consistent with multifinality.

We also examined the extent to which adolescent HED trajectory class could independently predict young adult alcohol outcomes while controlling for the antecedents of trajectory class membership. Once adjusting for the key antecedents, significant differences in the likelihood of binge drinking, getting drunk, and experiencing problems associated with alcohol use remained between those in the Increasing HED group compared to the None/Rare HED group. Thus, adolescent HED appeared to increase risk for harmful alcohol use at age 31 above and beyond risk and protective factors from respondents’ childhood and adolescence. HED in adolescence may therefore have unique predictive effects on adult alcohol use, and the adult alcohol outcomes were not solely due to the ongoing impacts of antecedents from earlier in life. This is encouraging from an early intervention standpoint – intervening on HED in adolescence may protect against the subsequent development of alcohol use problems later in life, even among individuals with early risk factors for alcohol use disorders (Hadland et al., 2019).

### Study contributions

The current study makes a number of contributions to the existing literature on HED during adolescence, and both its causes and consequences. We acknowledge that many findings from the current study align with past research. However, it is noteworthy that our results are quite consistent with prior research because of the unique characteristics of the RYDS sample. To our knowledge, the current study is the first to use GBTM to examine HED from a life-course perspective using a sample that comprises a majority of racially and socioeconomically minoritized youth, includes data from dyads of both adolescents and parents, and followed participants from early adolescence into their early 30s. The current study also examined a unique collection of antecedents to trajectory membership that spanned a broad period of early development. While other studies have importantly used GBTM to examine alcohol use in diverse cohorts of youth (e.g., Finlay et al., 2012; Flory et al., 2006), much prior GBTM research on HED during adolescence has relied on samples of mostly White, middle-class youth. This is a broader concern in substance use and prevention research, where much of what is known about the risk factors for adolescent substance use comes from research on White youth (e.g., Brown et al., 2004; Evans et al., 2017; Wallace & Muroff, 2002), yet there is evidence that adolescent alcohol use and alcohol-related problems vary by race/ethnicity and socioeconomic status (Chung et al., 2018; Johnston et al., 2015; Zemore et al., 2018).

An additional strength of the current study is that RYDS prospectively measured alcohol use up until the average respondent was 31 years old. Past research suggests that age 30 represents a point when most US adults begin to “age out” of harmful alcohol use (e.g., Lee et al., 2013). By analyzing alcohol use when the average RYDS respondent was 31, our research design allowed us to identify individuals who have not yet “aged out” of these behaviors, and who are therefore at risk of more durable and severe alcohol problems. Relatively few longitudinal studies of adolescent HED have measured alcohol use past age 30, representing another contribution of the current study. Moreover, much research on the etiology of persistent substance use has used predominantly White, clinical samples (e.g., Evans et al., 2017). Due to the characteristics of the RYDS sample, the current study helps to fill this gap in the literature on the developmental origins of alcohol problems in adulthood among a racially and socioeconomically diverse sample.

### Study limitations

Results should be interpreted in the context of some limitations. First, RYDS represents a unique cohort of adolescents who were in 7^th^ or 8^th^ grade in the 1987–1988 school year and living in one urban locale. These individuals grew up in a time of heightened adolescent substance use of all types, including alcohol (Levy et al., 2018), and it is likely that their experience in navigating adolescence and moving into adulthood in the context of substance use is different than contemporary youths. Therefore, the extent to which these findings are generalizable to other cohorts of young people is unknown. Also, although rates of attrition were quite low for a study that spanned nearly two decades, some 20% of participants did not participate in the phase 3 (adult) interview. In order to appropriately handle missing data, we utilized appropriate multiple imputation procedures.

Another important limitation pertains to the measures. We relied exclusively on self-report measures, and social-desirability bias may have affected our findings. In particular, the length of recall time for the early childhood adversity factors (measured when respondents were in early adulthood), as well as social-desirability bias, could have impacted the accuracy of these variables. We also note that alcohol researchers have noted the importance of defining HED or binge drinking differently for males and females in recent years. However, given the timing of our study, our measures did not adhere to these new recommendations. Likewise, although we examined a variety of alcohol measures in adulthood, we did not incorporate measures of alcohol use disorder using current diagnostic criteria. Our measures of the antecedents also did not cover all important factors. For example, we did not include a measure of genetic risk for alcohol use disorder. Additionally, measuring HED as a binary indicator, as opposed to a frequency, could have made it difficult to detect more nuanced HED patterns in GBTM. Lastly, several constructs were measured as single-item variables due to the breadth of the RYDS survey instrument and time constraints of the interview procedure. Multi-item scales would allow for more rigorous evaluation and handling of potential measurement error.

Additionally, because methods to handle missing data in the Stata Traj procedure are unavailable and it cannot adjust for covariates when assessing the effect of trajectory group membership on distal outcomes, we employed a “classify-analyze” approach to relate our trajectory groups to antecedents and outcomes. Although our posterior probabilities of class membership were reasonably high, latent trajectories are probabilistic and unobserved (Nagin & Tremblay, 2005). Therefore, assigning participants to observed trajectories with the “classify-analyze” approach may have produced biased estimates (Vermunt, 2010). However, sensitivity analyses shown in the appendix that do not rely on probabilistic classification (but suffer from other problems outlined earlier such as missing data due to listwise deletion) give us confidence in our results. Still, as with all studies, we recommend replicating and extending these analyses using various operationalizations of study variables, samples, and analytic procedures that address the research questions of interest in order to bolster the robustness and generalizability of our results.

Furthermore, while GBTM overcomes many challenges in analyzing longitudinal data, these models have some inherent limitations (Nagin & Tremblay, 2005). Latent trajectories represent average trends for subsamples of participants and do not account for individual differences in participant’s HED patterns (Nagin & Tremblay, 2005). Relatedly, GBTM often discerns latent trajectories that are characterized by “high,” “low,” and “increasing and/or decreasing” trends, suggesting that individual participants who had more variable or nuanced HED trends may not be well represented by our identified latent classes (Sher et al., 2011). Latent trajectories can also be overextracted when indicators are non-normal, as is the case with the HED variable in the current study (Bauer & Curran, 2003).

Last, while the diversity of our sample is a major benefit, the small number of White participants precluded our ability to estimate and compare the findings across racial/ethnic groups. The intersection between racial and socioeconomic disadvantages may importantly compound disparities in substance use (Pinedo, 2019; Zemore et al., 2018). Future research that investigates interactions between race and socioeconomic status may shed light on how the intersectionality of multiple minoritized identities influences alcohol-related problems. Accounting for gender and sexual identities in future HED research will also be important. Further, substance use rates across racial and ethnic groups can be nested within neighborhoods (Miech et al., 2020). However, the RYDS study was not designed to estimate contextual, multilevel effects. There are few children in each neighborhood of Rochester in our sample, and many children moved from one neighborhood to another over the course of the study, precluding reliable multilevel or nested analyses.

## Conclusions

These limitations notwithstanding, the present study provides a comprehensive developmental approach to understanding the etiology and consequences of HED among a racially and economically diverse cohort of adolescents, which is an understudied population in adolescent alcohol use literature. Our results extend previous research by replicating GBTM HED trajectories in a sample of predominantly Black youth from lower socioeconomic status backgrounds, examining an extensive collection of antecedents to trajectory membership from across early development, as well as evaluating long-term impacts of adolescent HED on alcohol use after emerging adulthood. Our findings identify a set of important antecedents that predict engagement in adolescent HED, many of which are modifiable and have been successful targets of intervention (e.g., delaying the onset of alcohol use, correcting normative perceptions of peer alcohol use, promoting pro-social peer groups, and treatment for parental alcohol use problems).

## Supplementary Material

Supplementary material

## Figures and Tables

**Table 1.** Distribution of G2 heavy episodic drinking by age (*N* = 939)

G2 age	*N*	Proportion reporting HED
14	343	0.02
14.5	549	0.02
15	688	0.03
15.5	804	0.07
16	840	0.10
16.5	777	0.12
17	655	0.13
17.5	505	0.15

*Note*. G2’s (focal adolescent) age at each wave was recorded to the nearest half year.

**Table 2.** Group-based trajectory model fit statistics by number of estimated latent groups (*N* = 939)

	Number of latent groups	
	2	3
AIC	−1574.1	−1569.6
BIC	−1594.1	−1596.6
*Avg. posterior probability of group membership*		
Group 1	0.95	0.62
Group 2	0.89	0.87
Group 3		0.87
*Predicted group membership (%)*		
Group 1	82.5	43.3
Group 2	17.5	44.2
Group 3		12.5
*Odds of correct classification*		
Group 1	4.4	2.2
Group 2	37.8	8.2
Group 3		46.4

**Table 3.** Model parameters for best-fitting group-based trajectory model (*N* = 939)

	Intercept	Linear	Quadratic	Group membership probability	Assigned group membership
Group 1: None/Rare HED	−17.084**	0.824**	–	0.825	0.847
Group 2: Increasing HED	−86.108**	9.930**	−0.285**	0.175	0.153

*Note*. Additional model diagnostics are in the Appendix. HED = heavy episodic drinking.

* *p* < .05;

** *p* < .01.

**Table 4.** Descriptive statistics for antecedents in the full sample and by latent trajectory group based on most likely class membership

Variable Set	Variables	Wave	*N* (full sample)	Type	Range	Full sample		Latent group 1: None/Rare HED		Latent group 2: Increasing HED	
						Mean/%	*SD*	Mean/%	*SD*	Mean/%	*SD*
*G2 HED trajectories*											
	Latent group 1: None/Rare HED	2–8	939	Binary	0,1	84.7					
	Latent group 2: Increasing HED	2–8	939	Binary	0,1	15.4					
*G2 demographics*											
	G2 Female	1	939	Binary	0,1	27.3		29.7		13.9	
	G2 White	1	939	Binary	0,1	15.3		14.3		20.8	
	G2 Hispanic	1	939	Binary	0,1	16.7		16.5		18.1	
	G2 Black	1	939	Binary	0,1	68.0		69.2		60.1	
	G2 Age at baseline	1	946	Continuous	11.4–15.5	13.9	0.8	13.9	0.8	14.1	0.8
	G2 Grade 7 at baseline	1	939	Binary	0,1	57.6		59.2		48.6	
	G2 Community arrest rate	1	939	Continuous	0.12–7.87	4.2	2.1	4.2	2.1	4.0	2.0
*Distal antecedents*											
Early life adversity	G1 Drank alcohol while pregnant	12	798	Binary	0,1	21.6		19.9		30.6	
	G1 Age at G2 birth	12	804	Continuous	13–45	22.7	5.6	22.9	5.7	21.6	4.8
	G2 Experienced neglect <12	12	842	Binary	0,1	8.2		7.0		15.3	
Early problems	G2 Drank alcohol <12	1	912	Binary	0,1	6.6		5.4		13.3	
	G2 Mental health symptoms <12	12	814	Binary	0,1	5.5		4.9		9.2	
*Proximate antecedents*											
Beliefs	G2 Alcohol beliefs	1	897	Continuous	1–4	3.6	0.7	3.7	0.6	3.2	0.9
	G2 Religiosity	1	897	Ordinal	1–5	2.5	1.0	2.6	1.1	2.1	0.8
	G2 School attachment	1	896	Continuous	1–4	3.1	0.3	3.1	0.3	3.0	0.4
Opportunities	G2 Time with peers	2	897	Ordinal	1–5	3.5	1.3	3.4	1.3	3.8	1.2
	G1 Supervision of G2	2	895	Continuous	1–4	3.7	0.6	3.7	0.5	3.5	0.7
	G2 Perceived access to alcohol	2	907	Ordinal	1–4	2.1	1.1	2.0	1.7	2.8	1.1
Social context	G1: Frequency of heavy drinking	2	878	Ordinal	0–3	0.5	0.8	0.5	0.8	0.8	0.9
	G2: Friend alcohol use	2	884	Ordinal	1–4	1.6	0.9	1.5	0.8	2.5	1.2

*Note*. For G2 alcohol beliefs, higher scores reflect beliefs that use is wrong. *SD* = standard deviation, G2 = generation 2 (the focal adolescent), G1 = generation 1 (G2’s primary caregiver), HED = heavy episodic drinking.

**Table 5.** Results of logistic regression analyses to predict latent class group membership for increasing frequency HED relative to none/rare HED (*N* = 939)

		Model 1		Model 2		Model 3	
Variable set	Variable	*OR*	95% CI	*OR*	95% CI	*OR*	95% CI
*Demographics*							
	G2 Female	0.414	(0.249, 0.689)	0.417	(0.248, 0.699)	0.353	(0.195, 0.637)
	G2 White (vs. Black)	1.532	(0.913, 2.571)	1.760	(1.014, 3.057)	1.507	(0.785, 2.893)
	G2 Hispanic (vs. Black)	1.147	(0.705, 1.866)	1.357	(0.807, 2.281)	1.958	(1.067, 3.594)
	G2 Community arrest rate	0.996	(0.903, 1.097)	1.012	(0.915, 1.120)	0.989	(0.881, 1.110)
	G2 Age at baseline	1.314	(0.964, 1.791)	1.313	(0.960, 1.795)	0.971	(0.679, 1.390)
	G2 Grade 7 at baseline	0.801	(0.509, 1.260)	0.821	(0.517, 1.304)	1.075	(0.625, 1.849)
*Distal antecedents*							
Early life adversity	G1 Drank alcohol while pregnant			1.691	(1.065, 2.684)	1.318	(0.753, 2.307)
	G1 Age at G2 birth			0.949	(0.910, 0.989)	0.947	(0.903, 0.993)
	G2 Experienced neglect <12			2.017	(1.111, 3.664)	1.654	(0.831, 3.290)
Early problems	G2 Drank alcohol <12			3.176	(1.696, 5.947)	1.571	(0.766, 3.220)
	G2 Mental health symptoms <12			1.218	(0.558, 2.660)	0.958	(0.383, 2.394)
*Proximate antecedents*							
Beliefs	G2 Alcohol beliefs					0.712	(0.529, 0.958)
	G2 Religiosity					1.201	(0.570, 2.531)
	G2 School attachment					0.676	(0.526, 0.871)
Opportunity factors	G2 Time with peers					1.190	(0.991, 1.430)
	G1 Supervision of G2					0.831	(0.585, 1.180)
	G2 Access to alcohol					1.335	(1.083, 1.646)
Social context	G1 Frequency of heavy drinking					1.423	(1.115, 1.818)
	G2 Friend alcohol use					2.189	(1.742, 2.752)

*Note*. Estimates are based on 20 multiply imputed data sets generated through chained equations. *OR* = odds ratio, CI = confidence interval, G2 = generation 2 (the focal adolescent), G1 = generation 1 (G2’s primary caregiver), HED = heavy episodic drinking.

**Table 6.** Descriptive statistics for adult alcohol outcome variables in the full sample and by latent trajectory group based on most likely class membership

Variables	Wave	*N* (full sample)	Type	Range	Full sample		Latent group 1: None/Rare HED		Latent group 2: Increasing HED	
					Mean/%	*SD*	Mean/%	*SD*	Mean/%	*SD*
Past Year: Monthly alcohol use	14	758	Binary	0,1	55.1		52.9		67.5	
Past Year: Binge drinking	14	758	Binary	0,1	29.0		25.5		49.1	
Past Year: Frequency binge drinking	14	758	Count	0–20	2.5	5.5	2.0	4.9	5.3	7.5
Past Year: Drunkenness	14	758	Binary	0,1	30.6		27.1		50.0	
Past Year: Frequency drunkenness	14	758	Count	0–10	1.5	3.0	1.2	2.7	3.2	4.1
Past Year: Alcohol-related problems	14	758	Binary	0,1	5.9		4.2		15.8	

*Note. SD* = standard deviation, G2 = generation 2 (the focal adolescent), G1 = generation 1 (G2’s primary caregiver), HED = heavy episodic drinking.

**Table 7.** Controlled effects of trajectory class membership on young adult alcohol use (*N* = 939)

Variable Set	Variable	Model 1		Model 2		Model 3		Model 4		Model 5		Model 6	
		Monthly alcohol use		Binge drinking		Frequency binge drinking		Drunkenness		Frequency of drunkenness		Alcohol-related problems	
		OR	95% CI	OR	95% CI	IRR	95% CI	OR	95% CI	IRR	95% CI	OR	95% CI
Trajectory	Increasing HED (vs. None/Rare HED)	1.501	(0.910, 2.476)	2.036	(1.282, 3.235)	1.978	(0.944, 4.142)	1.894	(1.178, 3.047)	1.742	(0.932, 3.256)	2.849	(1.277, 6.358)
Demographics	G2 Female	0.562	(0.394, 0.802)	0.289	(0.186, 0.450)	0.238	(0.138, 0.411)	0.377	(0.251. 0.567)	0.276	(0.173, 0.439)	0.380	(0.133, 1.088)
	G2 White (vs. Black)	1.023	(0.610, 1.715)	2.125	(1.274, 3.545)	1.882	(0.875, 4.047)	1.761	(1.050, 2.955)	1.797	(0.961, 3.359)	1.439	(0.562, 3.687)
	G2 Hispanic (vs. Black)	0.894	(0.595, 1.343)	1.277	(0.800, 2.037)	1.216	(0.648, 2.284)	1.062	(0.659, 1.712)	0.912	(0.555, 1.497)	1.698	(0.693, 4.160)
	G2 Community arrest rate	1.007	(0.934, 1.086)	1.004	(0.914, 1.103)	0.999	(0.887, 1.126)	1.086	(0.990, 1.192)	1.064	(0.954, 1.186)	1.001	(0.848, 1.182)
	G2 Age at baseline	0.956	(0.744, 1.229)	0.819	(0.604, 1.110)	0.936	(0.628, 1.397)	0.762	(0.569, 1.021)	0.777	(0.571, 1.057)	0.670	(0.412, 1.089)
	G2 Grade 7 at baseline	0.918	(0.612, 1.377)	0.969	(0.629, 1.494)	1.436	(0.757, 2.723)	0.795	(0.522, 1.210)	0.893	(0.565, 1.412)	1.080	(0.461, 2.528)
*Distal antecedents*													
Early life adversity	G1 Drank alcohol while pregnant	1.075	(0.722, 1.601)	0.938	(0.604, 1.458)	0.879	(0.447, 1.729)	1.045	(0.693, 1.576)	1.044	(0.604, 1.803)	1.313	(0.594, 2.899)
	G1 Age at G2 birth	1.008	(0.977, 1.040)	1.005	(0.973, 1.038)	0.998	(0.958, 1.040)	1.026	(0.994, 1.059)	1.023	(0.985, 1.062)	0.985	(0.916, 1.060)
	G2 Experienced neglect < 12	0.714	(0.402, 1.267)	1.029	(0.496, 2.135)	1.293	(0.486, 3.444)	0.779	(0.373, 1.629)	1.152	(0.586, 2.262)	1.051	(0.327, 3.380)
Early problems	G2 Drank alcohol < 12	0.637	(0.343, 1.184)	0.852	(0.436, 1.666)	1.256	(0.495, 3.185)	0.995	(0.523, 1.894)	1.285	(0.588, 2.807)	0.483	(0.111, 2.107)
	G2 Mental health symptoms < 12	0.723	(0.366, 1.427)	0.837	(0.398, 1.760)	0.517	(0.181, 1.472)	0.879	(0.421, 1.837)	0.570	(0.227, 1.427)	0.463	(0.095, 2.251)
*Proximate antecedents*													
Beliefs	G2 Alcohol beliefs	1.034	(0.806, 1.327)	1.002	(0.771, 1.300)	0.977	(0.653, 1.460)	1.048	(0.798, 1.377)	1.064	(0.764, 1.482)	0.868	(0.541, 1.391)
	G2 Religiosity	1.240	(0.761, 2.021)	1.202	(0.643, 2.250)	1.154	(0.533, 2.496)	1.315	(0.741, 2.333)	0.865	(0.468, 1.600)	0.540	(0.174, 1.676)
	G2 School attachment	0.903	(0.775, 1.053)	0.908	(0.759, 1.085)	0.972	(0.740, 1.278)	0.875	(0.739, 1.036)	0.924	(0.739, 1.154)	0.905	(0.607, 1.349)
Opportunity factors	G2 Time with peers	0.926	(0.813, 1.054)	0.902	(0.790, 1.029)	0.935	(0.777, 1.127)	0.947	(0.831, 1.080)	0.943	(0.809, 1.099)	0.939	(0.714, 1.235)
	G1 Supervision of G2	0.811	(0.618, 1.066)	0.900	(0.674, 1.201)	0.914	(0.608, 1.374)	0.756	(0.565, 1.011)	0.800	(0.584, 1,.095)	0.785	(0.479, 1.286)
	G2 Access to alcohol	1.174	(1.006, 1.371)	1.166	(0.975, 1.395)	1.150	(0.898, 1.471)	1.287	(1.072, 1.546)	1.238	(1.038, 1.467)	1.201	(0.869, 1.662)
Social context	G1 Frequency of heavy drinking	0.996	(0.819, 1.212)	0.977	(0.779, 1.225)	1.125	(0.836, 1.513)	0.959	(0.770, 1.194)	1.170	(0.904, 1.516)	1.354	(0.937, 1.955)
	G2 Friend alcohol use	1.095	(0.890, 1.348)	1.180	(0.959, 1.452)	1.324	(0.903, 1.942)	1.145	(0.927, 1.414)	1.180	(0.881, 1.579)	0.891	(0.603, 1.318)

*Note*. Estimates are based on 20 multiply imputed data sets. Each of the six models correspond to a different outcome variable. Monthly Alcohol Use, Binge Drinking, Drunkenness, and Alcohol Problems were estimated using a logit function. Frequency of Binge Drinking and Frequency of Drunkenness were estimated using a negative binomial distribution due to overdispersion. *OR* = odds ratio, *IRR* = incident rate ratio, CI = confidence interval, G2 = generation 2 (the focal adolescent), G1 = generation 1 (G2’s primary caregiver), HED = heavy episodic drinking.

## Footnotes

1 When the interview asked G2 respondents how much alcohol they “usually” drank, response options were: (1) I don’t drink; (2) One drink or less; (3) Two or three drinks; (4) Four or five drinks; (5) Six or more drinks. Because these response options combine “four or five drinks” into a single category, we used this threshold in our measure of HED.

2 It is well documented that the transition from middle school to high school is associated with an increase in adolescent alcohol use (Jackson & Schulenberg, 2013). However, given the nature (high-risk) and the level of grade retention and school dropout among the sample (e.g., 34 have dropped out of school in wave 4, 138 have dropped out of school in wave 5), grade level should not be used as a time metric to generate latent classes or groups because it is not always a time-changing (or applicable) measure. Nonetheless, age at wave 1 and year in school at wave 1 were included as antecedents to trajectory membership. Thus, we controlled for the relations between childhood and early adolescent grade retention and the transition to high school with patterns of HED in adolescence.

3 Within GBTM, the relations between antecedent variables and group membership can be estimated. It should be noted, though, that GBTM uses listwise deletion when estimating the model with antecedents. As a result, our analytic sample for assessing antecedents to latent class membership within the GBTM framework was 641. These results are available in Appendix Table B and are similar in nature and significance to those presented in the text that rely on a classify-analyze approach, but utilize all cases via multiple imputation of missing data.

4 A classify and analyze approach involves classifying each individual into their most probable class, and then performing subsequent analysis as though latent class membership is known (i.e., an observed categorical variable). This is not ideal as latent class analysis is a probability-based model that naturally takes uncertainty in classification into account, and a classify-analyze approach can bias the effect estimates in the subsequent analyses (Vermunt, 2010).

5 A two-group solution was also identified when using growth mixture models in MPlus (Muthén & Muthén, 2022), indicating a two-group solution is robust across software methodologies.

## References

[R1] American Psychiatric Association (2013). Diagnostic and statistical manual of mental disorders (5th ed.). American Psychiatric Publishing. 10.1176/appi.books.9780890425596

[R2] Bauer DJ, & Curran PJ (2003). Distributional assumptions of growth mixture models: Implications for overextraction of latent trajectory classes. Psychological Methods, 8(3), 338–363. 10.1037/1082-989X.8.3.338 14596495

[R3] Blanton H, Gibbons FX, Gerrard M, Conger KJ, & Smith GE (1997). Role of family and peers in the development of prototypes associated with substance use. Journal of Family Psychology, 11(3), 271–288. 10.1037/0893-3200.11.3.271

[R4] Brame B, Nagin DS, & Tremblay RE (2001). Developmental trajectories of physical aggression from school entry to late adolescence. Journal of Child Psychology and Psychiatry, 42(4), 503–512. 10.1111/1469-7610.00744 11383966

[R5] Brooks-Russell A, Simons-Morton B, Haynie D, Farhat T, & Wang J (2014). Longitudinal relationship between drinking with peers, descriptive norms, and adolescent alcohol use. Prevention Science, 15(4), 497–505. 10.1007/s11121-013-0391-9 23564529 PMC4160800

[R6] Brown TL, Miller JD, & Clayton RR (2004). The generalizability of substance use predictors across racial groups. The Journal of Early Adolescence, 24(3), 274–302. 10.1177/0272431604265677

[R7] Brown TL, Parks GS, Zimmerman RS, & Phillips CM (2001). The role of religion in predicting adolescent alcohol use and problem drinking. Journal of Studies on Alcohol, 62(5), 696–705. 10.15288/jsa.2001.62.696 11702809

[R8] Brunborg GS, Norström T, & Storvoll EE (2018). Latent developmental trajectories of episodic heavy drinking from adolescence to early adulthood: Predictors of trajectory groups and alcohol problems in early adulthood as outcome. Drug and Alcohol Review, 37(3), 389–395. 10.1111/dar.12565 28556439

[R9] Capaldi DM, Stoolmiller M, Kim HK, & Yoerger K (2009). Growth in alcohol use in at-risk adolescent boys: Two-part random effects prediction models. Drug and Alcohol Dependence, 105(1–2), 109–117. 10.1016/j.drugalcdep.2009.06.013 19625141 PMC2752270

[R10] Centers for Disease Control and Prevention (2022). Alcohol and public health. Department of Health and Human Services. https://www.cdc.gov/alcohol/index.htm

[R11] Chartier KG, Scott DM, Wall TL, Covault J, Karriker-Jaffe KJ, Mills BA, & Arroyo JA (2014). Framing ethnic variations in alcohol outcomes from biological pathways to neighborhood context. Alcoholism: Clinical and Experimental Research, 38(3), 611–618. 10.1111/acer.12304 24483624 PMC3959254

[R12] Chassin L, Pitts SC, & Prost J (2002). Binge drinking trajectories from adolescence to emerging adulthood in a high-risk sample: Predictors and substance abuse outcomes. Journal of Consulting and Clinical Psychology, 70(1), 67–78. 10.1037/0022-006X.70.1.67 11860058

[R13] Chung T, Creswell KG, Bachrach R, Clark DB, & Martin CS (2018). Adolescent binge drinking: Developmental context and opportunities for prevention. Alcohol Research: Current Reviews, 39(1), 5–15. 30557142 10.35946/arcr.v39.1.02 PMC6104966

[R14] Chung T, & Jackson KM (2019). Adolescent alcohol use. In Zucker R, & Brown S (Eds.), The Oxford handbook of adolescent substance abuse (pp. 1–70). Oxford University Press. 10.1093/oxfordhb/9780199735662.013.007

[R15] Cicchetti D (1999). A developmental psychopathology perspective on drug abuse. In Glantz MD, & Hartel CR (Eds.), Drug abuse: Origins & interventions (pp. 97–117). American Psychological Association. 10.1037/10341-005

[R16] Cicchetti D, & Rogosch FA (1996). Equifinality and multifinality in developmental psychopathology. Development and Psychopathology, 8(4), 597–600. 10.1017/S0954579400007318

[R17] Cicchetti D, & Rogosch FA (2002). A developmental psychopathology perspective on adolescence. Journal of Consulting and Clinical Psychology, 70(1), 6–20. 10.1037/0022-006X.70.1.6 11860057

[R18] D’Amico EJ, Tucker JS, Miles JN, Ewing BA, Shih RA, & Pedersen ER (2016). Alcohol and marijuana use trajectories in a diverse longitudinal sample of adolescents: Examining use patterns from age 11 to 17 years. Addiction, 111(10), 1825–1835. 10.1111/add.13442 27130360 PMC5016216

[R19] Donovan JE (2019). Child and adolescent socialization into substance use. In Zucker RA, & Brown SA (Eds.), The Oxford handbook of adolescent substance abuse. Oxford University Press. 10.1093/oxfordhb/9780199735662.013.018

[R20] Donovan JE, & Molina BS (2013). Types of alcohol use experience from childhood through adolescence. Journal of Adolescent Health, 53(4), 453–459. 10.1016/j.jadohealth.2013.03.024 PMC3783556 23763961

[R21] Evans EA, Grella CE, Washington DL, & Upchurch DM (2017). Gender and race/ethnic differences in the persistence of alcohol, drug, and poly-substance use disorders. Drug and Alcohol Dependence, 174, 128–136. 10.1016/j.drugalcdep.2017.01.021 28324815

[R22] Faggiano F, Vigna-Taglianti F, Burkhart G, Bohrn K, Cuomo L, Gregori D, Panella M, Scatigna M, Siliquini R, Varona L, van der Kreeft P, Vassara M, Wiborg G, & Galanti MR (2010). The effectiveness of a school-based substance abuse prevention program: 18-month follow-up of the EU-Dap cluster randomized controlled trial. Drug and Alcohol Dependence, 108(1–2), 56–64. 10.1016/j.drugalcdep.2009.11.018 20080363

[R23] Fairbairn CE, Briley DA, Kang D, Fraley RC, Hankin BL, & Ariss T (2018). A meta-analysis of longitudinal associations between substance use and interpersonal attachment security. Psychological Bulletin, 144(5), 532–555. 10.1037/bul0000141 29494194 PMC5912983

[R24] Finlay AK, White HR, Mun EY, Cronley CC, & Lee C (2012). Racial differences in trajectories of heavy drinking and regular marijuana use from ages 13 to 24 among African-American and white males. Drug and Alcohol Dependence, 121(1–2), 118–123. 10.1016/j.drugalcdep.2011.08.020 21908109 PMC3258364

[R25] Flory K, Brown TL, Lynam DR, Miller JD, Leukefeld C, & Clayton RR (2006). Developmental patterns of African American and Caucasian adolescents’ alcohol use. Cultural Diversity and Ethnic Minority Psychology, 12(4), 740–746. 10.1037/1099-9809.12.4.740 17087533

[R26] Fortier CB, Whitworth JW, Fonda JR, Currao A, Beck BM, Levin L, Esterman M, Milberg WP, & McGlinchey RE (2021). Early adolescent binge drinking increases risk of psychopathology in post-9/11 veterans and mild traumatic brain injury exacerbates symptom severity. Alcohol and Alcoholism, 56(1), 116–124. 10.1093/alcalc/agaa075 32776121

[R27] Gatzke-Kopp LM (2016). Diversity and representation: Key issues for psychophysiological science. Psychophysiology, 53(1), 3–13. 10.1111/psyp.12566 26681612

[R28] Goldschmidt L, Richardson GA, De Genna NM, Cornelius MD, & Day NL (2019). Prenatal alcohol exposure and offspring alcohol use and misuse at 22 years of age: A prospective longitudinal study. Neurotoxicology and Teratology, 71, 1–5. 10.1016/j.ntt.2018.11.001 30399401 PMC6330135

[R29] Hadland SE, & Baer TE (2014). The racial and ethnic gap in substance use treatment: Implications for US healthcare reform. Journal of Adolescent Health, 54(6), 627–628. 10.1016/j.jadohealth.2014.03.015 24726462

[R30] Hadland SE, Knight JR, & Harris SK (2019). Alcohol use disorder: A pediatric-onset condition needing early detection and intervention. Pediatrics, 143(3), e20183654. 10.1542/peds.2018-3654 30783023 PMC6398366

[R31] Hawkins JD, Catalano RF, Morrison DM, O’Donnell J, Abbott RD, & Day LE (1992). The Seattle Social Development Project: Effects of the first four years on protective factors and problem behaviors. In McCord J, & Tremblay RE (Eds.), Preventing antisocial behavior: Interventions from birth through adolescence (pp. 139–161). Guilford Press.

[R32] Henry KL, & Slater MD (2007). The contextual effect of school attachment on young adolescents’ alcohol use. Journal of School Health, 77(2), 67–74. 10.1111/j.1746-1561.2007.00169.x 17222157

[R33] Hicks BM, Iacono WG, & McGue M (2010). Consequences of an adolescent onset and persistent course of alcohol dependence in men: Adolescent risk factors and adult outcomes. Alcoholism: Clinical and Experimental Research, 34(5), 819–833. 10.1111/j.1530-0277.2010.01154.x 20184563 PMC2884045

[R34] Hill KG, White HR, Chung IJ, Hawkins JD, & Catalano RF (2000). Early adult outcomes of adolescent binge drinking: Person-and variable-centered analyses of binge drinking trajectories. Alcoholism: Clinical and Experimental Research, 24(6), 892–901. 10888080 PMC1847635

[R35] Hingson RW, Heeren T, & Winter MR (2006). Age at drinking onset and alcohol dependence: Age at onset, duration, and severity. Archives of Pediatrics & Adolescent Medicine, 160(7), 739–746. 10.1001/archpedi.160.7.739 16818840

[R36] Hoeben EM, Rulison KL, Ragan DT, & Feinberg ME (2021). Moderators of friend selection and influence in relation to adolescent alcohol use. Prevention Science, 22(5), 567–578. 10.1007/s11121-021-01208-9 33709307 PMC8229127

[R37] Jackson KM, & Schulenberg JE (2013). Alcohol use during the transition from middle school to high school: National panel data on prevalence and moderators. Developmental Psychology, 49(11), 2147–2158. 10.1037/a0031843 23421801 PMC3933211

[R38] Jennings WG (2015). Life-course/developmental theories. In Jennings WG(Ed.), The encyclopedia of crime and punishment. Wiley-Blackwell. 10.1002/9781118519639.wbecpx152

[R39] Johnston LD, O’Malley PM, Miech RA, Bachman JG, & Schulenberg JE (2015). Demographic subgroup trends among adolescents in the use of various licit and illicit drugs, 1975–2014. Monitoring the future occasional paper series. Paper 83. Institute for Social Research. https://hdl.handle.net/2027.42/137897

[R40] Keyes KM, Schulenberg JE, O’Malley PM, Johnston LD, Bachman JG, Li G, & Hasin D (2012). Birth cohort effects on adolescent alcohol use: The influence of social norms from 1976 to 2007. Archives of General Psychiatry, 69(12), 1304–1313. 10.1001/archgenpsychiatry.2012.787 22868751 PMC3597448

[R41] Kuntsche E, Kuntsche S, Thrul J, & Gmel G (2017). Binge drinking: Health impact, prevalence, correlates and interventions. Psychology & Health, 32(8), 976–1017. 10.1080/08870446.2017.1325889 28513195

[R42] Lee MR, Chassin L, & Villalta IK (2013). Maturing out of alcohol involvement: Transitions in latent drinking statuses from late adolescence into adulthood. Development and Psychopathology, 25, 1137–1153. 10.1017/S0954579413000424 24229554 PMC3831177

[R43] Levy S, Campbell MD, Shea CL, & DuPont R (2018). Trends in abstaining from substance use in adolescents: 1975–2014. Pediatrics, 142(2), e20173498. 10.1542/peds.2017-3498 30026244

[R44] Lewis MA, & Neighbors C (2006). Social norms approaches using descriptive drinking norms education: A review of the research on personalized normative feedback. Journal of American College Health, 54(4), 213–218. 10.3200/JACH.54.4.213-218 16450845 PMC2459316

[R45] Little RJ, & Rubin DB (2019). Statistical analysis with missing data (Vol. 793). John Wiley & Sons.

[R46] Masten AS, Roisman GI, Long JD, Burt KB, Obradović J, Riley JR, Boelcke-Stennes K, & Tellegen A (2005). Developmental cascades: Linking academic achievement and externalizing and internalizing symptoms over 20 years. Developmental Psychology, 41(5), 733–746. 10.1037/0012-1649.41.5.733 16173871

[R47] Mayes LC, & Suchman NE (2006). Developmental pathways to substance abuse. In Cicchetti D, & Cohen DJ (Eds.), Developmental psychopathology: Risk, disorder, and adaptation (pp. 599–619). John Wiley & Sons. 10.1002/9780470939406.ch16

[R48] Mewton L, Lees B, & Rao RT (2020). Lifetime perspective on alcohol and brain health. BMJ, 371(1), m4691. 10.1136/bmj.m4691 33272963

[R49] Miech RA, Johnston LD, O’Malley PM, Bachman JG, Schulenberg JE, & Patrick ME (2020). Monitoring the future national survey results on drug use, 1975–2019: Volume I, Secondary school students. Institute for Social Research, The University of Michigan. http://monitoringthefuture.org/pubs.html#monographs

[R50] Moffitt TE (1993). Adolescence-limited and life-course-persistent antisocial behavior: A developmental taxonomy. Psychological Review, 100(4), 674–701. 10.1037/0033-295X.100.4.674 8255953

[R51] Moure-Rodríguez L, & Caamano-Isorna F (2020). We need to delay the age of onset of alcohol consumption. International Journal of Environmental Research and Public Health, 17(8), 2739. 10.3390/ijerph17082739 32316106 PMC7215939

[R52] Mulia N, Karriker-Jaffe KJ, Witbrodt J, Bond J, Williams E, & Zemore SE (2017). Racial/ethnic differences in 30-year trajectories of heavy drinking in a nationally representative US sample. Drug and Alcohol Dependence, 170(1), 133–141. 10.1016/j.drugalcdep.2016.10.031 27889594 PMC5270645

[R53] Muthén LK, & Muthén BO (1998-2022). Mplus user’s guide. Muthén & Muthén. https://www.statmodel.com/ugexcerpts.shtml

[R54] Nagin DS (2005). Group-based modeling of development. Harvard University Press. http://www.jstor.org/stable/j.ctvjf9z1f

[R55] Nagin DS, & Odgers CL (2010a). Group-based trajectory modeling in clinical research. Annual Review of Clinical Psychology, 6(1), 109–138. 10.1146/annurev.clinpsy.121208.131413 20192788

[R56] Nagin DS, & Odgers CL (2010b). Group-Based trajectory modeling (nearly) two decades later. Journal of quantitative criminology, 26(4), 445–453. 10.1007/s10940-010-9113-7 21132047 PMC2994902

[R57] Nagin DS, & Tremblay RE (2005). Developmental trajectory groups: Fact or a useful statistical fiction? Criminology, 43(4), 873–904. 10.1111/j.1745-9125.2005.00026.x

[R58] National Institute on Alcohol Abuse and Alcoholism (2021, February 25). What is a Standard Drink? https://www.niaaa.nih.gov/alcohols-effects-health/overview-alcohol-consumption/what-standard-drink

[R59] National Institute on Drug Abuse. (2022, January 17). Monitoring the Future Study: Trends in Prevalence of Various Drugs. https://www.drugabuse.gov/drug-topics/trends-statistics/monitoring-future/monitoring-future-study-trends-in-prevalence-various-drugs

[R60] Oesterle S, Hill KG, Hawkins JD, Guo JIE, Catalano RF, & Abbott RD (2004). Adolescent heavy episodic drinking trajectories and health in young adulthood. Journal of Studies on Alcohol, 65(2), 204–212. 10.15288/jsa.2004.65.204 15151351 PMC1876676

[R61] Olsson CA, Romaniuk H, Salinger J, Staiger PK, Bonomo Y, Hulbert C, & Patton GC (2016). Drinking patterns of adolescents who develop alcohol use disorders: Results from the Victorian Adolescent Health Cohort Study. BMJ Open, 6(2), e010455. 10.1136/bmjopen-2015-010455 PMC4762151 26868948

[R62] Osgood DW, Wilson JK, O’Malley PM, Bachman JG,&Johnston LD (1996). Routine activities and individual deviant behavior. American Sociological Review, 61(4), 635–655. 10.2307/2096397

[R63] Pamplin JR, Susser ES, Factor-Litvak P, Link BG, & Keyes KM (2020). Racial differences in alcohol and tobacco use in adolescence and mid-adulthood in a community-based sample. Social Psychiatry and Psychiatric Epidemiology, 55(4), 457–466. 10.1007/s00127-019-01777-9 31542795 PMC7083697

[R64] Park E, McCoy TP, Erausquin JT, & Bartlett R (2018). Trajectories of risk behaviors across adolescence and young adulthood: The role of race and ethnicity. Addictive Behaviors, 76, 1–7. 10.1016/j.addbeh.2017.07.014 28734192

[R65] Patrick ME, Schulenberg JE, Maggs JL, & Maslowsky J (2014). Substance use and peers during adolescence and emerging/early adulthood: Socialization, selection, and developmental transitions. In Sher K (Ed.), Handbook of substance use disorders. Oxford University Press. 10.1093/oxfordhb/9780199381678.013.004

[R66] Pinedo M (2019). A current re-examination of racial/ethnic disparities in the use of substance abuse treatment: Do disparities persist? Drug and Alcohol Dependence, 202, 162–167. 10.1016/j.drugalcdep.2019.05.017 31352305 PMC10676029

[R67] Roeder K, Lynch KG, & Nagin DS (1999). Modeling uncertainty in latent class membership: A case study in criminology. Journal of the American Statistical Association, 94(447), 766–776.

[R68] Rossow I, Keating P, Felix L, & McCambridge J (2016). Does parental drinking influence children’s drinking? A systematic review of prospective cohort studies. Addiction, 111(2), 204–217. 10.1111/add.13097 26283063 PMC4832292

[R69] Rubin DB (1987). Multiple imputation for nonresponse in surveys. Wiley. 10.1002/9780470316696,

[R70] Santrock JW (2009). Life-span development (12th ed.). McGraw Hill.

[R71] Schulenberg JE, & Maggs JL (2008). Destiny matters: Distal developmental influences on adult alcohol use and abuse. Addiction, 103(s1), 1–6. 10.1111/j.1360-0443.2008.02172.x 18426536

[R72] Schulenberg JE, Patrick ME, Maslowsky J, & Maggs JL (2014). The epidemiology and etiology of adolescent substance use in developmental perspective. In Lewis M, & Rudolph K (Eds.), Handbook of developmental psychopathology (3rd ed., pp. 601–620). Springer.

[R73] Sher KJ, Jackson KM, & Steinley D (2011). Alcohol use trajectories and the ubiquitous cat’s cradle: Cause for concern? Journal of Abnormal Psychology, 120(2), 322–335. 10.1037/a0021813 21319874 PMC3091989

[R74] Spadoni AD, McGee CL, Fryer SL, & Riley EP (2007). Neuroimaging and fetal alcohol spectrum disorders. Neuroscience & Biobehavioral Reviews, 31(2), 239–245. 10.1016/j.neubiorev.2006.09.006 17097730 PMC1820628

[R75] Squeglia LM, & Gray KM (2016). Alcohol and drug use and the developing brain. Current Psychiatry Reports, 18(5), 1–10. 10.1007/s11920-016-0689-y 26984684 PMC4883014

[R76] Staff J, Whichard C, Siennick SE, & Maggs J (2015). Early life risks, antisocial tendencies, and preteen delinquency. Criminology, 53(4), 677–701. 10.1111/1745-9125.12093 26900167 PMC4755357

[R77] StataCorp 2021). Stata statistical software: Release 17. StataCorp LLC.

[R78] Steinberg L (2008). A social neuroscience perspective on adolescent risk-taking. Developmental Review, 28(1), 78–106. 10.1016/j.dr.2007.08.002 18509515 PMC2396566

[R79] Stoltzfus JC (2011). Logistic regression: a brief primer. Academic Emergency Medicine, 18(10), 1099–1104. 10.1111/j.1553-2712.2011.01185.x 21996075

[R80] Substance Abuse and Mental Health Services Administration (2021). Key substance use and mental health indicators in the United States: Results from the 2020 National Survey on Drug Use and Health (HHS Publication No. PEP21-07-01-003, NSDUH Series H-56). Center for Behavioral Health Statistics and Quality, Substance Abuse and Mental Health Services Administration. https://www.samhsa.gov/data/

[R81] Sudhinaraset M, Wigglesworth C, & Takeuchi DT (2016). Social and cultural contexts of alcohol use: Influences in a social-ecological framework. Alcohol Research: Current Reviews, 38(1), 35–45. 27159810 10.35946/arcr.v38.1.05 PMC4872611

[R82] Thornberry TP, Ireland TO, & Smith CA (2001). The importance of timing: The varying impact of childhood and adolescent maltreatment on multiple problem outcomes. Development and Psychopathology, 13(4), 957–979. 10.1017/S0954579401004114 11771916

[R83] Tucker JS, Ellickson PL, Orlando M, Martino SC, & Klein DJ (2005). Substance use trajectories from early adolescence to emerging adulthood: A comparison of smoking, binge drinking, and marijuana use. Journal of Drug Issues, 35(2), 307–332. 10.1177/002204260503500205

[R84] Tucker JS, Orlando M, & Ellickson PL (2003). Patterns and correlates of binge drinking trajectories from early adolescence to young adulthood. Health Psychology, 22(1), 79–87. 10.1037/0278-6133.22.1.79 12558205

[R85] Vermunt JK (2010). Latent class modeling with covariates: Two improved three-step approaches. Political Analysis, 18(4), 450–469. 10.1093/pan/mpq025

[R86] Wallace JM, & Muroff JR (2002). Preventing substance abuse among African American children and youth: Race differences in risk factor exposure and vulnerability. Journal of Primary Prevention, 22(3), 235–261. 10.1023/A:1013617721016

[R87] Walters GD (2014). Drugs, crime, and their relationships: Theory, research, practice, and policy. Jones & Bartlett Publishers.

[R88] Wilcox P (2003). An ecological approach to understanding youth smoking trajectories: Problems and prospects. Addiction, 98, 57–77. 10.1046/j.1360-0443.98.s1.5.x 12752362

[R89] Windle M (2010). A multilevel developmental contextual approach to substance use and addiction. BioSocieties, 5(1), 124–136. 10.1057/biosoc.2009.9 22754585 PMC3384512

[R90] Witbrodt J, Mulia N, Zemore SE, & Kerr WC (2014). Racial/ethnic disparities in alcohol-related problems: Differences by gender and level of heavy drinking. Alcoholism: Clinical and Experimental Research, 38(6), 1662–1670. 10.1111/acer.12398 24730475 PMC4047188

[R91] Yuen WS, Chan G, Bruno R, Clare P, Mattick R, Aiken A, Boland V, McBride N, McCambridge J, Slade T, & Kypri K (2020). Adolescent alcohol use trajectories: Risk factors and adult outcomes. Pediatrics, 146(4), e20200440. 10.1542/peds.2020-0440 32968030

[R92] Zapolski TCB, Pedersen SL, McCarthy DM, & Smith GT (2014). Less drinking, yet more problems: Understanding African American drinking and related problems. Psychological Bulletin, 140(1), 188–223. 10.1037/a0032113 23477449 PMC3758406

[R93] Zemore SE, Karriker-Jaffe KJ, Mulia N, Kerr WC, Ehlers CL, Cook WK, Martinez P, Lui C, & Greenfield TK (2018). The future of research on alcohol-related disparities across US racial/ethnic groups: A plan of attack. Journal of Studies on Alcohol and Drugs, 79(1), 7–21. 10.15288/jsad.2018.79.7 29227222 PMC5894859

