# Supplementary material for: Heavy episodic drinking in adolescence and alcohol-related problems in adulthood: A developmental approach to alcohol use across the life course
Source: Dev Psychopathol. Author manuscript; Available in PMC 2025 May 27. (PMC12108369; doi:10.1017/S0954579422001249)
Supplement: Supplementary material [file NIHMS2081660-supplement-Supplementary_material.docx]

**Appendix**

**Additional Information on Measures Included in Final Models**

Below we provide additional detail on select measures retained in final models.

***Early Life Adversity***

The wave 12 interview was administered to both G1 and G2 respondents about 9 years after the first RYDS interview, when the average G2 respondent was around 23 years old. A unique aspect of the wave 12 interview for both G1s and G2s is that it contained a collection of retrospective items designed to measure G2’s early life circumstances, including questions about potentially sensitive topics such as maternal substance use during pregnancy and G2 experiences with child abuse.

In the G2 wave 12 interview, G2s were asked a series of questions related to neglect. For example, G2 respondents were asked whether neighbors had ever fed them or cared for them because their parents/caretaker had neglected to purchase groceries; whether they had spent the night with a neighbor because no one was taking care of them at home; and whether they experienced circumstances where they were not being given enough to eat, kept clean enough, or getting medical care that they needed. At the end of this section, G2 respondents were asked the following question: “Looking back on when you were a child younger than 12, do you now feel that there were circumstances where you were neglected?” *G2 experienced neglect < age 12* is a binary indicator coded 1 if the G2 respondent answered “yes” to this question, and 0 otherwise.

***Beliefs***

During the wave 1 interview, G2 respondents were asked to rate the extent to which they agreed with 14 statements regarding their experiences with school. The statements addressed topics such as the respondent’s perception of their own academic ability (e.g., “I do poorly at school”), interest in schoolwork (e.g., “I try hard at school”), investment in earning good grades (e.g., “Getting good grades is important to me”), and feelings toward teachers (e.g., “I don’t care what teachers think of me”). Response options ranged from 1 (“Strongly Disagree”) to 4 (“Strongly Agree”). After coding all items such that greater values corresponded to higher levels of school attachment, we found that the 14 items demonstrated relatively high internal consistency (α = .79). *G2 school attachment* represents each G2 respondent’s mean response across all 14 items.

***G2 Alcohol Use in Adulthood***

We operationalized G2 alcohol use by creating six variables based on questions asked at the wave 14 interview. Collectively, these six variables intended to capture a wide variety of drinking behaviors, ranging from relatively innocuous alcohol use (e.g., drinking at least once per month) to higher-risk alcohol use (e.g., getting drunk). Notably, subsequent questions about alcohol use were only asked to participants who previously indicated they had consumed alcohol at least once per month in the past year.

G2 respondents were asked: “During the past year, did you drink beer, wine, wine coolers, or liquor at least once a month?” *Drank alcohol at least once per month* is a binary indicator coded 1 if the G2 respondent answered “yes” to this question, and 0 otherwise. Respondents who answered “yes” to this item were then asked: “During the past year, have you had five or more drinks at one sitting?” *Consumed 5 or more drinks in one sitting* is a binary indicator coded 1 if the G2 respondent answered “yes” to this question, and 0 otherwise. If the G2 respondent indicated that they had consumed five or more drinks at one sitting during the previous year, the interview asked the following open-ended question: “About how many times have you done this during the past year?” Upon reviewing the original frequency distribution for this item, we found that most respondents reported engaging in this behavior 20 or fewer times, though a small percentage of respondents reported extremely high values. To avoid potential issues related to influential outliers, we top-coded this item so that the maximum value was set to 20. *Number of times consumed 5+ drinks* is a count variable that represents how G2 answered this question. Respondents who previously indicated that they did not consume five or more drinks in one sitting were coded as 0.

The wave 14 interview also asked G2 respondents: “During the past year, have you gotten drunk?” *Drunkenness* is a binary indicator coded 1 if respondents answered “yes” to this question, and 0 otherwise. If the G2 respondent indicated drunkenness in the previous year, the interview asked the following open-ended question: “About how many times have you done this during the past year?” As before, we reviewed the original frequency distribution and decided to top-code this item so that the maximum value was set to 10. *Frequency of Drunkenness* is a count variable that represents how G2 answered this question. Respondents who previously indicated that they did not get drunk in the previous year were coded as 0.

Finally, G2 respondents who indicated that they drank alcohol at least once a month in the past year were asked whether they had experienced various problems as a result of drinking alcohol. Eligible respondents were presented with a series of 10 yes/no questions that asked whether G2 had experienced a variety of problems in the past year as a direct result of drinking alcohol. The questions addressed a host of topics, including potential signs of alcohol dependence (e.g., “Have you tried to cut down on drinking and found that you could not?”); physical aggression (e.g., “Have you gotten into physical fights because of drinking?”); driving while intoxicated (e.g., “Have you had your driver’s license suspended for driving under the influence of alcohol?”); relationship problems (e.g., “Have you had problems with your friends because of drinking?”); challenges meeting role obligations (e.g., “Have you gotten into trouble at school or work because of drinking?”); and health issues (e.g., “Have you had problems with your health because of drinking?”). *Alcohol-related problems* is a binary indicator coded 1 if the G2 respondent answered “yes” to at least one of these 10 items, and 0 otherwise.

**Antecedent Measures Not Included in Final Models**

Below we describe antecedent variables not retained in final models after variable selection steps (see Results).

***G1 Demographics***

We constructed measures of basic background characteristics for the G1 (parent/caretaker) respondents based on their responses during the wave 1 interview. *G1 married* is a binary indicator coded 1 if the G1 respondent reported they were married, and 0 otherwise. *G1 full time employment* is a binary indicator coded 1 if the G1 respondent reported they were employed and worked at least 40 hours in a normal week, and 0 otherwise. *G1 received welfare benefits* is a binary indicator coded 1 if the G1 respondent reported their household had received government assistance in the previous 6 months (e.g., food stamps, public assistance, Aid for Dependent Children), and 0 otherwise.

***Early Life Adversity***

During the wave 12 interview, G1 was asked whether G2 weighed less than 5 pounds at birth. *G2 low birth weight* is a binary indicator coded 1 if G1 answered “yes,” and 0 otherwise. G1 was also asked if they smoked cigarettes while pregnant with G2. *G2’s biological mother smoked cigarettes while pregnant* is a binary indicator coded 1 for “yes” and 0 for “no.”

***Early Problems***

During the Wave 12 interview, G1 respondents were also asked whether G2 ever had “attention deficit disorder,” “other learning disabilities,” or “mental retardation.” If G1 answered “yes” to any of these three items, they were asked how old G2 was when this challenge was first noticed. *G2 cognitive symptoms < age 12* is a binary indicator coded 1 if G1 reported that G2 experienced one or more of these three challenges before age 12, and 0 otherwise.

During the wave 1 interview, when G2 respondents were about age 13.5, G2 respondents were asked whether they had ever engaged in a variety of delinquent or antisocial behaviors. If the G2 respondent indicated that they had engaged in the behavior, they were asked: “How old were you the first time you did this?” *G2 shoplifted < age 12* is a binary indicator of whether G2 reported that they shoplifted for the first time before age 12 (1 = shoplifted prior to age 12, 0 = did not shoplift prior to age 12). *G2 assault < age 12* follows the same coding scheme based on whether G2 reported they “hit someone with the idea of hurting them” before age 12.

***Beliefs***

During the wave 1 interview, G2 respondents were asked: “Of all the teachers you have known, how many have you liked?” Response options ranged from 0 (“None of them”) to 4 (“All of them”). *G2 teacher positive relations with teachers* is an ordinal measure that represents how respondents answered this question.

***Social Context***

In addition to being asked about friends’ alcohol use, during the wave 2 interview G2 was asked “About how many kids in your neighborhood drink alcohol without their parents' permission?” Response options ranged from 1 (“None”) to 4 (“A lot”), and *Number of kids in G2’s neighborhood who drank alcohol* is an ordinal measure based on this response scale. *G2 perception of inconsistent punishment* is an ordinal variable from the wave 2 interview based on G2’s response to: “How often do you get punished for something at one time, and then at other times not get punished for the same thing?” Participants responded based on their experience with G1 and values ranged from 1 (“Never”) to 4 (“Often”).

**Appendix Tables**

| Appendix Table A. Group-based Trajectory Model Diagnostics | | | | |
| --- | --- | --- | --- | --- |
| Latent Group | Group Membership Probability and 95% Confidence Interval | Proportion Assigned to Group | Average Posterior Probability of Group Membership | Odds Correct Classification |
| None/Rare HED | 0.825 (0.784, 0.866) | 0.847 | 0.954 | 4.4 |
| Increasing HED | 0.175 (0.134, 0.216) | 0.153 | 0.889 | 37.8 |
| *Note.* Statistical diagnostics that allow for classify and analyze are in line with recommendations by Nagin (2005) for model adequacy, including tight 95% CI for group membership, close alignment between group membership probability and proportion assigned to group, and the average posterior probability for group membership above .7 (per recommendations of Roerder et al., 1999). HED = heavy episodic drinking. | | | | |

| Appendix Table B. Odds Ratios and 95% Confidence Intervals for Antecedents Predicting Latent Trajectory Membership using GBTM (*N* = 641) | | | |
| --- | --- | --- | --- |
|  |  | *OR* | 95% CI |
| Demographics | G2 Female | 0.310 | (0.146, 0.656) |
|  | G2 White (vs. Black) | 1.153 | (0.493, 2.695) |
|  | G2 Hispanic (vs. Black) | 1.849 | (0.849, 4.026) |
|  | G2 Community arrest rate | 0.976 | (0.846, 1.127) |
|  | G2 Age at baseline | 0.946 | (0.607, 1.474) |
|  | G2 Grade 7 at baseline | 1.142 | (0.577, 2.259) |
| Distal Predictive Factors |  |  |  |
| Early Life Adversity | G1 Drank alcohol while pregnant | 1.142 | (0.616, 2.117) |
|  | G1 Age at birth | 0.951 | (0.902, 1.003) |
|  | G2 Experienced neglect < 12 | 1.908 | (0.839, 4.336) |
| Early Problems | G2 Drank alcohol < 12 | 1.484 | (0.655, 3.365) |
|  | G2 Mental health symptoms < 12 | 1.430 | (0.535, 3.818) |
| Proximate Predictive Factors |  |  |  |
| Beliefs | G2 Alcohol beliefs | 0.653 | (0.454, 0.938) |
|  | G2 Religiosity | 1.038 | (0.428, 2.519) |
|  | G2 School attachment | 0.659 | (0.489, 0.888) |
| Opportunity Factors |  |  |  |
|  | G2 Time with peers | 1.264 | (1.009, 1.582) |
|  | G1 Supervision of G2 | 0.743 | (0.494, 1.117) |
|  | G2 Access to alcohol | 1.373 | (1.065, 1.772) |
| Social Context |  |  |  |
|  | G1 Frequency of heavy drinking | 1.613 | (1.209, 2.153) |
|  | G2 Friend alcohol use | 1.899 | (1.440, 2.055) |
| *Note.* Estimates are from the TRAJ procedure taking into account class membership uncertainty. Effects represent the odds of belonging to the Increasing HED trajectory relative to the None/Rare HED trajectory. *N* = 641 due to use of listwise deletion for missing data in the GBTM. *OR* = odds ratio, CI = confidence interval, HED = heavy episodic drinking. | | | |
